# Biphenyl Modulates the Expression and Function of Respiratory Oxidases in the Polychlorinated-Biphenyls Degrader *Pseudomonas pseudoalcaligenes* KF707

**DOI:** 10.3389/fmicb.2017.01223

**Published:** 2017-06-30

**Authors:** Federica Sandri, Stefano Fedi, Martina Cappelletti, Francesco M. Calabrese, Raymond J. Turner, Davide Zannoni

**Affiliations:** ^1^Department of Pharmacy and Biotechnology, University of BolognaBologna, Italy; ^2^Department of Biosciences, Biotechnology and Pharmacological Sciences, University of Bari “Aldo Moro”Bari, Italy; ^3^Department of Biology, University of Bari “Aldo Moro”Bari, Italy; ^4^Department of Biological Sciences, University of CalgaryCalgary, AB, Canada

**Keywords:** biphenyl growth, gene expression, respiratory activities, terminal oxidases, *Pseudomonas pseudoalcaligenes* KF707

## Abstract

*Pseudomonas pseudoalcaligenes* KF707 is a soil bacterium which is known for its capacity to aerobically degrade harmful organic compounds such as polychlorinated biphenyls (PCBs) using biphenyl as co-metabolite. Here we provide the first genetic and functional analysis of the KF707 respiratory terminal oxidases in cells grown with two different carbon sources: glucose and biphenyl. We identified five terminal oxidases in KF707: two *c(c)aa*_3_ type oxidases (Caa_3_ and Ccaa_3_), two *cbb*_3_ type oxidases (Cbb_3_1 and Cbb_3_2), and one *bd* type cyanide-insensitive quinol oxidase (CIO). While the activity and expression of both Cbb_3_1 and Cbb_3_2 oxidases was prevalent in glucose grown cells as compared to the other oxidases, the activity and expression of the Caa_3_ oxidase increased considerably only when biphenyl was used as carbon source in contrast to the Cbb_3_2 oxidase which was repressed. Further, the respiratory activity and expression of CIO was up-regulated in a Cbb_3_1 deletion strain as compared to W.T. whereas the CIO up-regulation was not present in Cbb_3_2 and C(c)aa_3_ deletion mutants. These results, together, reveal that both function and expression of *cbb*_3_ and *caa*_3_ type oxidases in KF707 are modulated by biphenyl which is the co-metabolite needed for the activation of the PCBs-degradation pathway.

## Introduction

The bacterium *Pseudomonas pseudoalcaligenes* KF707, isolated in the 1980s near a biphenyl (BP) manufacturing plant in Japan (Furukawa and Miyazaki, 1986), is known as one of the most effective aerobic degraders of polychlorinated biphenyls (PCBs) (Fedi et al., 2001). Notably, these harmful and highly hydrophobic organic compounds are not primary substrates for cell growth so that a substrate such as biphenyl is required to support growth and induction of the PCBs degradation pathway (Furukawa and Miyazaki, 1986; Furukawa et al., 1993; Fedi et al., 2001). In this way, bacteria such as KF707 that are able to grow on biphenyl have the capacity to co-metabolize various PCBs congeners (Fedi et al., 2001). Despite the fact that most of the biphenyl degradation enzymes required for PCBs degradation are expressed during the aerobic growth of KF707 (Furukawa et al., 1993), no report is available on the functional arrangement of KF707 respiratory chain with biphenyl as carbon source. Indeed, an early biochemical study was published which aimed to define the effect of the toxic oxyanion tellurite on aerobic growth of KF707 in rich-medium (LB-broth) (Di Tomaso et al., 2002). This work suggested the presence in KF707 of a branched respiratory chain leading to two cytochrome (cyt) *c* oxidases and one cyanide insensitive quinol oxidase (CIO) (Di Tomaso et al., 2002). Since then no other studies on function and composition of the respiratory redox chain of this aerobic PCBs degrader have been published while in the meantime the expression and arrangement of the respiratory chains in species such as *Pseudomonas* (*P*.) *putida* PAK and *P. aeruginosa* PAO1, were elucidated (Kawakami et al., 2010; Arai, 2011; Sevilla et al., 2013; Arai et al., 2014). In strain PAO1 it was shown that the *aa*_3_ type oxidase, which plays a major role in aerobic respiration of several bacterial species (Poole and Cook, 2000), is expressed primarily under nutrient-limited conditions and is otherwise a minor player under nutrient-rich growth conditions, e.g., LB-broth (Kawakami et al., 2010). Conversely, two *cbb*_3_ type oxidases (Cbb_3_1 and Cbb_3_2) and a cyanide insensitive oxidase (CIO) are crucial when oxygen becomes limiting and during growth in biofilms (Comolli and Donohue, 2002, 2004; Alvarez-Ortega and Harwood, 2007; Kawakami et al., 2010). The Cbb_3_1 oxidase is expressed constitutively while the Cbb_3_2 is induced through oxygen limitation (Comolli and Donohue, 2004; Kawakami et al., 2010). Notably, mutation of the *cco1* genes coding for the Cbb_3_1 oxidase causes up-regulation of the CIO promoter in *P. aeruginosa* (Kawakami et al., 2010) indicating that the interactive regulation of the genes for Cbb_3_-1 and CIO is mediated by the redox-sensitive transcriptional regulator RoxSR (Bueno et al., 2012).

To fill the molecular and functional gap between our present knowledge of respiration in KF707 and the most investigated *Pseudomonas* spp., here we provide the first functional analysis of the KF707 respiratory genes (GenBank Acc. no. AP014862; Triscari-Barberi et al., 2012) in relation with two different primary carbon sources for growth such as glucose and biphenyl, while the remaining oxidative growth conditions were kept constant. This is an important aspect as the response of the central carbon catabolism to nutritional compounds is an absolute requirement for effective microbial colonization of a given environment (Rojo, 2010; Shimizu, 2014). Nevertheless, the modulation by the growth carbon source of the oxidative electron transport chain (ETC) that is the machinery which transduces into energy the reducing metabolic power, remains a puzzling issue (Dominguez-Cuevas et al., 2006; Nikel and Chavarria, 2015; Nikel et al., 2016).

Here we show that KF707 contains five different aerobic terminal oxidases: two of *c(c)aa*_3_ type (Caa_3_ and Ccaa_3_), two isoforms of *cbb*_3_ type (Cbb_3_1 and Cbb_3_2), and one *bd* type cyanide-insensitive quinol oxidase (CIO). However, while the function and expression of both Cbb_3_1 and Cbb_3_2 oxidases is prevalent in glucose grown cells as compared to other oxidases, the expression of Caa_3_ and Cbb_3_2 oxidases were 4-fold increased and 7-fold decreased, respectively, when biphenyl was used as the sole carbon source along with a very low contribution to respiration of CIO. Furthermore, the respiratory activity and expression of CIO in glucose grown cells were up to 7 times higher in Cbb_3_1 deletion mutant as compared to W.T. cells whereas this CIO up-regulation was not present in Cbb_3_2 and C(c)aa_3_ deletion mutants.

This work not only reveals unexpected features of *P. pseudoalcaligenes* KF707 respiratory chain such as for example the presence of a Caa_3_ oxidase induced by growth in biphenyl but it also integrates the functional and genetic data obtained in the past with this PCBs-degrader (Taira et al., 1992; Furukawa et al., 1993; Fujihara et al., 2006; Tremaroli et al., 2007, 2008, 2010, 2011) allowing the establishment of solid molecular basis to better understand the use of toxic aromatics as energy and carbon source.

## Materials and methods

### Bacterial strains and growth conditions

*P. pseudoalcaligenes* KF707, wild type (W.T.) and mutants, and *Escherichia coli* strains harboring cloning vectors and recombinant plasmids used in this study are described in Table 1. Luria Bertani medium (LB), pH 7.2, was routinely used for bacterial growth. Trans-conjugants were selected on AB defined medium, pH 7.2, (K_2_HPO_4_, 3 g L^−1^; NaH_2_PO_4_, 1 g L^−1^; NH_4_Cl, 1 g L^−1^; MgSO_4_, 300 mg L^−1^; KCl 150 mg L^−1^, CaCl_2_, 10 mg L^−1^; FeSO_4_ 7H_2_O 2.5 mg L^−1^) with D-glucose (5 g L^−1^) as carbon source. Antibiotics were added to KF707 and *E. coli* growth medium at the following concentrations: ampicillin (Amp), 50 μg mL^−1^, kanamycin (Km), 25 μg mL^−1^, gentamycin (Gm), 10 μg mL^−1^.

**Table 1:** List of bacterial strains and plasmids, used in this study.

**Bacterial Strains**	**Relevant genotype**	**Source or References**
***P. pseudoalcaligenes*** **KF707**		
*Wild type* (W.T.)	Amp^r^	Furukawa and Miyazaki, 1986
KFΔcox1	Deletion of *coxI-II-III*, Amp^r^	This study
KFΔcox2	Deletion of *coxMNOP*, Amp^r^	This study
KFΔcox1-2	Deletion of *coxI-II-III* and *coxMNOP*, Amp^r^	This study
KFΔcco1	Deletion of *ccoN1O1Q1P1*, Amp^r^	This study
KFΔcco2	Deletion of *ccoN2O2Q2P2*, Amp^r^	This study
KFΔcco1-2	Deletion of *ccoN1O1Q1P1* and *ccoN2O2Q2P2*, Amp^r^	This study
KFΔcox1-2/cco1-2	Deletion of *coxI-II-III, coxMNOP, ccoN1O1Q1P1*, and *ccoN2O2Q2P2*, Amp^r^	This study
KFΔCIO	Deletion of *cioABC*, Amp^r^	This study
KFΔcox1-2/CIO	Deletion of *coxI-II-III, coxMNOP* and *cioABC*, Amp^r^	This study
KFLac	KF707 *lacZ* with no insertion, Amp^r^	This study
KFcox1Lac	KF707 *coxII::lacZ* translational fusion, Amp^r^	This study
KFcox2Lac	KF707 *coxM::lacZ* translational fusion, Amp^r^	This study
KFcco1Lac	KF707 *ccoN1::lacZ* translational fusion, Amp^r^	This study
KFcco2Lac	KF707 *cco*N2*::lacZ* translational fusion, Amp^r^	This study
KFCIOLac	KF707 *cioA::lacZ* translational fusion, Amp^r^	This study
KFΔcco1-2 CIOLac	Deletion of *ccoN1O1Q1P1* and *ccoN2O2Q2P2, cioA::lacZ* translational fusion, Amp^r^	This study
KFΔcox1-2/cco1-2 CIOLac	Deletion of *coxI-II-III, coxMNOP, ccoN1O1Q1P1* and *ccoN2O2Q2P2, cioA::lacZ* translational fusion Amp^r^	This study
KFΔcco1 CIOLac	Deletion of *ccoN1O1Q1P1, cioA::lacZ* translational fusion, Amp^r^	This study
KFΔcco2 CIOLac	Deletion of *ccoN2O2Q2P2, cioA::lacZ* translational fusion, Amp^r^	This study
***Escherichia coli***		
DH5α	*sup*E44, *hsd*R17, *rec*A1, *end*A1, *gyr*A96, *thi*1, *rel*A1	Hanahan, 1983
HB101	Sm^r^, *rec*A, *thi, pro, leu, hsd*R	Boyer and Roulland-Dussoix, 1969
**Plasmids**	**Relevant genotype**	**Source or Reference**
pRK2013	Km^r^, *ori* ColE1, RK2-Mob+, Rka-Tra+	Figurski and Helinski, 1979
pG19II	Gm^r^, *sac*B, *lac*Z, cloning vector and conjugative plasmid	Maseda et al., 2004
pG19IIΔcox1	Gm^r^, *sac*B, *lac*Z, carrying Δcox1 (*coxI-II-III*) deleted fragment	This study
pG19IIΔcox2	Gm^r^, *sac*B, *lac*Z, carrying Δcox2 (*coxMNOP*) deleted fragment	This study
pG19IIΔcco1	Gm^r^, *sac*B, *lac*Z, carrying Δcco1 (*ccoN1O1Q1P1*) deleted fragment	This study
pG19IIΔcco2	Gm^r^, *sac*B, *lac*Z, carrying Δcco2 (*ccoN2O2Q2P2*) deleted fragment	This study
pG19IIΔCIO	Gm^r^, *sac*B, *lac*Z, carrying ΔCIO (*cioABC*) deleted fragment	This study
pTNS3	RK6 replicon, encodes the TnsABC+D specific transposition pathway, helper plasmid DNA, Amp^r^	Choi et al., 2005
pFLP2-Km	Flp recombinase-expressing plasmid, Ap^r^, Km^r^	Hoang et al., 1998
pUC18-mini-Tn*7*T-Gm-*lacZ*	mini-Tn*7*, for construction of β-galactosidase protein fusions, *lacZ*, Gm^r^	Choi and Schweizer, 2006
pUC-cox1-lacZ	Mini-Tn*7*T, *coxII::lacZ* translational fusion, Gm^r^	This study
pUC-cox2-lacZ	Mini-Tn*7*T, *coxM::lacZ* translational fusion, Gm^r^	This study
pUC-cco1-lacZ	Mini-Tn*7*T, *ccoN1::lacZ* translational fusion, Gm^r^	This study
pUC-cco2-lacZ	Mini-Tn*7*T, *ccoN2::lacZ* translational fusion, Gm^r^	This study
pUC-CIO-lacZ	Mini-Tn*7*T, *cioA::lacZ* translational fusion, Gm^r^	This study

*Names of KF707's deletion and translational fusion mutant strains were assigned based on Kawakami et al. (2010)*.

Growth curves of KF707 W.T. and mutant strains on different carbon sources (glucose and biphenyl) were conducted in 250 mL Erlenmeyer flasks containing 50 mL of mineral salt medium (MSM), pH 7.2, at 30°C and 130 rpm (Tremaroli et al., 2010). A single colony of KF707 was first inoculated in 10 mL of LB medium and grown overnight, cells were centrifuged and washed twice with 0.1 M phosphate buffer (pH 7.0) then inoculated (1.0% v/v) on MSM medium supplemented with a single carbon source (biphenyl or glucose). Each carbon source was added to the sterile medium in order to have a final carbon concentration of 6 mM. Growth curves were performed by monitoring the OD_600_ level, every 2 h, until the late stationary phase (24 h for growth with glucose and 30 h for growth with biphenyl) was reached. Generation times (*g*) were calculated during the exponential growth phase. A one-way ANOVA was performed to test the null hypothesis that were no differences in the mean of g of the strains (see Text for details), followed by a two-sample *T*-test within pairs of strains.

### Construction of KF707 terminal cytochrome oxidase deleted mutants

Nucleotide and amino acid sequences used in this study were based on the complete genome sequence of *P. pseudolcaligenes* KF707 available in GenBank under the accession no. AP014862. Sequence similarity searches were performed using BLAST software (https://blast.ncbi.nlm.nih.gov/Blast.cgi) (Altschul et al., 1990) together with the conserved domain database (https://www.ncbi.nlm.nih.gov/Structure/cdd/wrpsb.cgi) while multiple sequence alignments were performed with ClustalW software (Thompson et al., 1994). The accession numbers of the genes mentioned in this paper are summarized in Table S2 of the Supplementary Material or directly reported in the text.

KF707 deleted mutants for single or multiple oxidases (Table 1) were obtained by Gene SOEing PCR technique (Izumi et al., 2007). For the construction of recombinant sequences with deletion in specific genes the primer pairs for the amplification of the upstream flanking region, and the primer pairs for the amplification of the downstream flanking region, were used (Table S1). The outer primers, specifically the forward for the upstream region and the reverse for the downstream one, were designed with specific restriction sites (Table S1). The inner primers, specifically the reverse for the upstream region and the forward for the downstream one, included overlapping sequences in order to join the flanking regions together as described previously (Izumi et al., 2007) (Table S1).

The recombinant sequences with the deletion genes obtained by the SOEing method, were double digested and cloned in specific restriction sites of the conjugative plasmid pG19II in order to construct pG19IIΔcox1, pG19IIΔcox2, pG19IIΔcco1, pG19IIΔcco2, and pG19IIΔCIO (Table 1) (Maseda et al., 2004). Recombinant plasmids were introduced into chemically competent cells of *E. coli* DH5α host and transformants were selected for Gm resistance along with white/blue screening by adding X-gal to agar media at a final concentration of 40 μg mL^−1^. *E. coli* DH5α, containing each pG19II recombinant plasmid, was used as donor strain and plasmid was transferred by tri-parental conjugation to the recipient KF707 wild type strain by means of the helper strain *E. coli* HB101 pRK2013. The conjugation included two steps of homologous recombination. The first crossover recombination results in integration of the recombined pG19II plasmid into the genome, transconjugants were selected for their resistance to Gm and sensitivity to sucrose (Maseda et al., 2004). After the second single crossover recombination, only the cells that had lost pG19II were selected by growth in the presence of 20% sucrose; their phenotype at the end result was Gm sensitive and sucrose resistance (Maseda et al., 2004). Deletion mutants were confirmed by PCR and sequencing analysis.

### Construction of KF707 lacZ translational fusion mutants

To evaluate the activity of terminal oxidases in KF707, β-galactosidase assays were performed by using mutant strains containing translational fusions of the terminal oxidases with *lacZ* gene. The *lacZ*-containing strains were constructed by utilizing a Tn*7*-based method and the translational fusion fragments were inserted into the genome of KF707 at the *att*Tn*7* site located downstream of *glmS* (Choi and Schweizer, 2006).

The promoter region, the ATG start codon and the coding region for the first 10 amino-acids, of *coxII* (*cox1* gene cluster—KFcox1Lac), *coxM* (*cox2* gene cluster—KFcox2Lac*), ccoN1* (*cco1* gene cluster—KFcco1Lac), *ccoN2* (*cco2* gene cluster—KFcco2Lac), and *cioA* (*cio* gene cluster—KFCIOLac) were amplified by PCR, using primers in the Table S1. These fragments were cloned into the pUC-miniTn*7*-Gm-*lacZ* vector and the resultant plasmid were electroporated, with the helper plasmid pTNS3 (Choi et al., 2005), into KF707. Then the mutant strains were obtained with the method described by Kawakami et al. (2010).

Finally, β-galactosidase assays were performed at 30°C in MSM minimal medium with glucose or biphenyl as single carbon source, at two different phases of growth (exponential phase—OD_600 nm_ 0.3–0.5 and stationary phase—OD_600 nm_ 0.7–0.9) using a standard protocol (Sambrook et al., 1989); each experiment was repeated at least six times.

### Enzymatic activities with NADI assay

To observe the cytochrome *c* oxidases activity in intact cells of W.T. and mutant strains, a NADI assay was carried out (Marrs and Gest, 1973). KF707 strains were grown until the stationary phase (OD_600 nm_ 0.7–0.9), in minimal medium (MSM) with glucose or biphenyl as single carbon source. 1 mL of culture was collected, centrifuged and washed twice with 1 ml of Tris-HCl 10 mM (pH 7.5). Subsequently, 100 μL of a 1:1 mixture of 35 mM α-naphthol, in ethanol, and 30 mM *N,N*-dimetyl-*p*-phenylenediamine monohydrochloride (DMPD), in water, was added to the cells. The reaction mixture was incubated at room temperature for a few seconds and the color change was observed within 1 min.

### Preparation of membrane fragments

For the preparation of membrane fragments, cells were grown aerobically until the stationary phase (OD_600 nm_ 0.7–0.9) in 3 L Erlenmeyer flasks containing 1 L of mineral salt medium (MSM), pH 7.2 at 30°C and 130 rpm, with 6 mM of glucose or biphenyl as single carbon source. Growths were stopped after 16 and/or 30 h for glucose or biphenyl medium, respectively. Cells were washed twice with 0.1 M phosphate buffer to reach about 1.6 g of wet weight cells. Membrane fragments for respiratory activities and spectroscopic analysis were obtained using French pressure cell and ultracentrifugation, in 50 mM MOPSO buffer (pH 7.2) containing 5 mM MgCl_2_ (Di Tomaso et al., 2002). Membranes were suspended at a known protein concentration (5–10 mg mL^−1^) in the same buffer and used immediately for analysis. Experiments were conducted in membranes of W.T. and mutant cells from at least two/three independent cell preparations (see Figure and Table legends).

### Spectroscopic analyses, respiratory activities, and protein determination

The amounts of cytochromes in membrane fragments were estimated by recording reduced (with 0.5 mM NADH plus 5 mM KCN and/or a few crystals of sodium dithionite)-*minus*-oxidized [with a few crystal(s) of potassium ferricyanide] optical difference spectra at room temperature with a Jasco 7800 spectrophotometer. Absorption coefficients ϵ_603−630_ of 11.6 mM^−1^ cm^−1^, ϵ_561−575_ of 22 mM^−1^ cm^−1^, and ϵ_551−542_ of 19.1 mM^−1^ cm^−1^ were used to determine the amounts of *a*-, *b*-, and *c*-type cytochromes, respectively (Kishikawa et al., 2010).

Respiratory activities in membrane fragments isolated from KF707 W.T. and deletion mutant strains, were determined by monitoring the oxygen consumption with a Clark-type oxygen electrode YSI 53 (Yellow Springs Instruments) as described previously (Daldal et al., 2001). Activities in the presence or absence of specific inhibitors (see Text for details) were measured within a few hours after the end of the membrane isolation procedure. The inhibitory concentration IC_50_ was the concentration of an inhibitor required to inhibit 50% of the target enzymatic activity.

Protein content of samples was determined using the Lowry assay with bovine serum albumin (BSA) as a standard (Lowry et al., 1951).

## Results

### Putative genes for terminal oxidases

#### The aa_3_ type cytochrome oxidases of KF707

**Figure 1:**
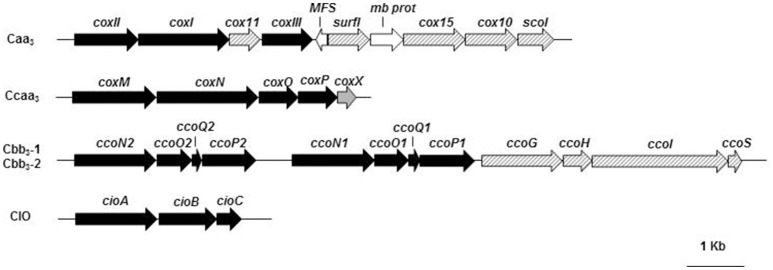
Organization of the terminal oxidase gene clusters in KF707: *cox*, encoding for cytochrome *c* oxidases of *caa*_3_type (*coxI-II-III*) and *ccaa*_3_type (*coxMNOP*); *cco*, encoding for gene products involved in the synthesis and assembly of two cytochrome *c* oxidases of *cbb*_3_type (*ccoN1O1Q1P1* and *ccoN2O2Q2P2*); *cio*, gene cluster for the quinol oxidase CIO (*cioABC*). Genes (listed in Table S2) are represented by arrows and by different colors or stripes: black, functional genes; dashed stripes, genes for cytochrome biogenesis; gray, cytochrome *c* oxidase accessory membrane protein (CoxX); white, genes supposedly not directly related with the gene clusters under analysis. Full names and abbreviations used: MFS, Major Facilitator Superfamily permease; mb prot, membrane protein.

The analysis of the *P. pseudoalcaligenes* KF707 complete genome shows the presence of two gene clusters for two different *cox* oxidases of *aa*_3_ type whose subunits are predicted to contain *c*-type hemes: *caa*_3_ and *ccaa*_3_ type oxidases. The *caa*_3_ type oxidase is encoded by *coxI-II-III* (BAU71738-71737-71740) gene cluster (Figure 1; Table S2). The three subunits, which are annotated as part of *aa*_3_ type cytochrome *c* oxidase in the KF707 genome, have sequences with high similarity (~90%) to CoxB, CoxA, and CoxC of *P. aeruginosa* (Stover et al., 2000). Similarly to *P. aeruginosa*, subunit I (58.8 KDa) of KF707 is predicted to carry a low spin *a*-type heme and one heme *a*_3_-Cu_B_ binuclear catalytic center. The predicted subunit II (42 KDa) consists of a membrane anchored cupredoxin domain, containing the electron-accepting homo-binuclear copper-center, Cu_A_, with a carboxy-terminal fusion to a cytochrome *c* domain (Lyons et al., 2012). The presence of a *c*-type heme in the *aa*_3_ type oxidase of KF707 was suggested by an alignment analysis (Supplementary Material—Alignment 1) which showed, in the C-terminal region, the typical amino-acids residues (Cxx-CH), that coordinate the heme *c* in *Thermus thermophilus* and *Rhodothermus marinus caa*_3_ oxidases (Lyons et al., 2012); these characteristic residues are absent from *Rhodobacter sphaeroides* and *Paracoccus denitrificans aa*_3_ oxidases while the CoxII amino acid sequence of KF707 has 81% similarity with the CoxB of *P. aeruginosa* PAO1.

In KF707 an additional *ccaa*_3_ type oxidase is predicted to be encoded by the gene cluster *coxMNOP* (BAU74428-74432) (Figure 1; Table S2). Sequence analyses showed that this alternative complex is characterized by the organization which is typical of *aa*_3_ type cytochrome oxidases present in *Sinorhizobium meliloti* 1021, *Cupriavidus metallidurans* CH34, *Mesorhizobium* sp., and *Polaromonas* (Preisig et al., 1996b). Previous studies have shown that the *coxMNOP* gene cluster encodes a complex with homology to Cu-containing cyt *c* oxidase; indeed it was observed that the subunit I (66 KDa), encoded by *coxN*, was very similar to CoxA of *P. aeruginosa* (Bott et al., 1992) and to CoxI of KF707's *caa*_3_. The alignment suggested that CoxM (52 KDa) of KF707 showed, in the C-terminal portion, two *c*-type hemes (Supplementary Material—Alignment 2) and therefore this oxidase is a *ccaa*_3_ cytochrome oxidase; in this case, in the C terminal portion, there is a repetition of the residues (Cxx-CH) that coordinate the *c*-type hemes. These enzymes are not common, but they have also been found in bacteria such as *Desulfovibrio vulgaris* (Lobo et al., 2008) and *Shewanella oneidensis* MR-1 (Le Laz et al., 2014). In KF707 CoxP and CoxO amino acid sequences are homologs to subunit III of the *aa*_3_ complex.

#### The cbb_3_ type oxidases of KF707

Two complete sets of genes encoding cyt *c* oxidases of *cbb*_3_ type (Cbb_3_1 and Cbb_3_2) are present in KF707 (Figure 1; Table S2). These genes, tandemly clustered in the genome of KF707, are situated in the *ccoN1O1Q1P1* (BAU73552-73555) and *ccoN2O2Q2P2* (BAU73556-73559) clusters as previously shown in *P. aeruginosa* strains PAO1 and PA7, *P. putida* KT2440, and *P. fluorescens* (Stover et al., 2000). Similarly to orthodox *cbb*_3_ type oxidases, the catalytic subunit I (~50 KDa) of KF707 encoded by *ccoN* gene is expected to comprise 12 transmembrane helices and to contain, in addition to the low spin *b*-type heme, a binuclear center formed by high spin *b*_3_ heme and Cu_B_. In general, the two trans-membrane cytochrome *c* subunits with *c*-type monoheme and diheme groups are encoded by the genes *ccoO* (~22 KDa) and *ccoP* (~40 KDa) respectively, while CcoQ (~7 KDa) are necessary for the stability of the complex. These oxidases utilize cytochrome *c* as an electron donor and they lack a Cu_A_ site.

In general, the *ccoNOQP* operons encoding *cbb*_3_ oxidase subunits are found in association with the *ccoGHIS* (BAU73548-73551) gene cluster which is located in the downstream region and whose expression is required for the maturation and assembly of a functional *cbb*_3_ oxidase and this type of gene organization is also present in KF707 (Figure 1; Table S2) (Preisig et al., 1996a; Koch et al., 2000; Pitcher and Watmough, 2004). Homology searching of both *ccoNOQP* and *ccoGHIS*, showed the same gene arrangement among *P. aeruginosa* strains PAO1 and PA7, *P. putida* KT2440, and *P. entomophila* L48 while *P. aeruginosa* strains UCBPP-PA14 and LESB58, *P. stutzeri* A1501, and *P. mendocina* YMP showed similar features except that some of them lack one copy of the *ccoQ* gene.

#### The cyanide-insensitive oxidase of KF707

In the past, an oxidase activity insensitive to cyanide (CIO), which accounts for ~20% of the total NADH-dependent respiration of LB-grown KF707 cells, has been reported (Di Tomaso et al., 2002). Here we show that KF707 genome contains two genes coding for two subunits which are highly similar to those of cytochrome *bd* type quinol oxidases of *Pseudomonas fulva 12-X* and *P. mendocina* (Figure 1; Table S2). In *E. coli* and other Gram negative bacteria, the cyt *bd*-I complex consists of two subunits named CydA (subunit I) and CydB (subunit II), while in *Pseudomonas* spp. it is encoded by the *cioA* and *cioB* genes, respectively (Cunningham et al., 1997). In KF707, genes *cioA* (BAU72498) and *cioB* (BAU72499) code for two orthodox subunits in addition to a third accessory subunit encoded by *cioC* (BAU72500).

The *bd* oxidase functions as a quinol oxidase with a relatively low sensitivity to cyanide and it shows often high affinity for oxygen (D'mello et al., 1996). CIO contains low-spin heme *b*_558_, high spin heme *b*_595_ and heme *d* (Jünemann, 1997; Siletsky et al., 2016). It should be noted that although both the *cioA* and *cioB* genes are highly similar to the *cydAB* genes encoding a *bd*-type oxidase, the CIO from KF707 lacks the spectral features of hemes *b*_595_ and heme *d* (Matsushita et al., 1983; Di Tomaso et al., 2002) (not shown).

### Expression of membrane bound oxidases

Early biochemical results have shown that membranes isolated from KF707 cells grown in LB-growth medium do not contain significant amounts of *aa*_3_ type hemes (Di Tomaso et al., 2002). However, since KF707 is capable to aerobically degrade PCBs in the presence of biphenyl as co-metabolite (Abramowicz, 1990) we thought to determine the expression of the entire set of the putative genes for terminal oxidases in cells grown in biphenyl as compared to cells grown on a defined carbon source such as glucose.

**Figure 2:**
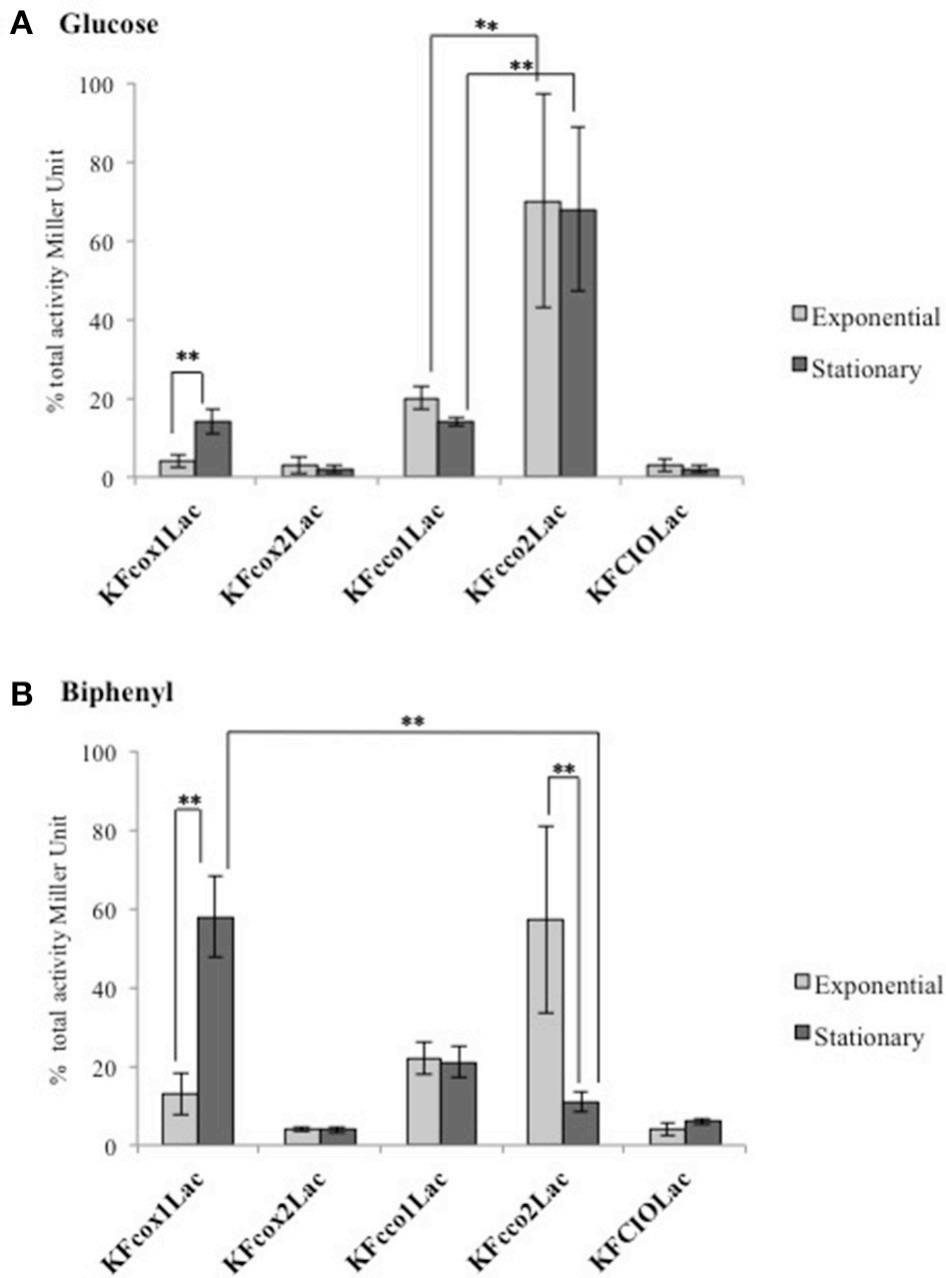
β-Galactosidase activities measured in cell extracts derived from the KF707 translational fusion mutant strains (Table 1), grown aerobically in MSM medium with glucose **(A)** and biphenyl **(B)** as sole carbon source. The assays were performed (at least six times) at two different stages of growth (exponential phase OD_600 nm_ 0.3–0.5 and stationary phase OD_600 nm_ 0.7–0.9) and the activities are presented as percentage of total activity. Error bars indicate standard deviation of the means. Asterisks indicate that mean values are significantly different according to one-way ANOVA and verified by a two samples *T*-test within pairs of strains (^**^p < 0.01).

The expression pattern in response to the carbon source was determined using *lacZ* translational fusions that monitor the promoter activities at the translational level. Figure 2 shows the β-galactosidase activity of each translational fusion when KF707 cells were cultured in glucose and biphenyl medium and harvested at the same cell density in their exponential and stationary growth phases.

The *cco1* promoter for the Cbb_3_1 exhibited similar basic level of expression in both exponential and stationary phases in cells grown with glucose (Figure 2A). Using the same carbon source, the *cco2* promoter for the Cbb_3_2 showed a high activity in both exponential and stationary phases of growth. In contrast to *cco2*, the *cox1* promoter for the Caa_3_ oxidase had a low activity in cells harvested at their exponential growth phase while an activity similar to that of the *cco1* promoter was seen in the stationary growth phase. The *cox2* promoter for the Ccaa_3_ and the *cio* promoter for the CIO oxidase showed very low activities in both exponential and/or stationary grown cells.

In cells grown with biphenyl (Figure 2B) the *cco1* promoter exhibited similar levels of expression in both exponential and stationary growth phases. In contrast to *cco1*, the *cco2* promoter showed a high expression level in the exponential phase while *cco2* expression was very low in the stationary phase of growth. Interestingly, the drop of *cco2* expression in stationary phase was matched with a drastic increase of *cox1* expression which was 4- and 10-times higher than that seen in the exponential growth phase on biphenyl and glucose, respectively. Similarly to cells grown in glucose, the *cox2* and the *cio* promoters of cells grown in biphenyl showed very low expression values in both exponential and stationary growth phases.

### Growth profiles and NADI assay

**Figure 3:**
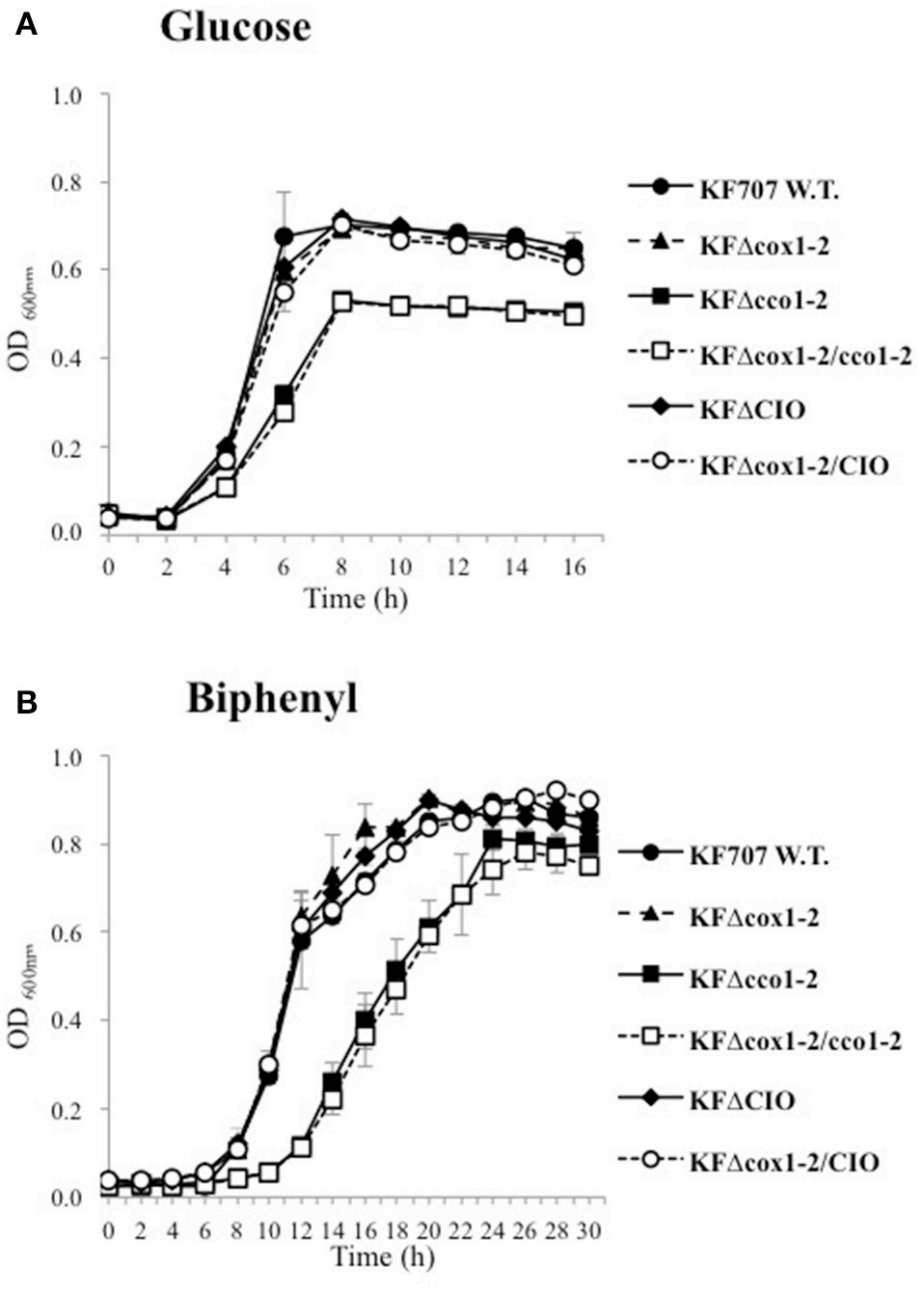
Growth curves of KF707 W.T. and deletion mutant strains (Table 1). Strains were grown in 50 mL of MSM medium in 250 mL flasks shaken at 130 r.p.m, with 6 mM of glucose **(A)** or biphenyl **(B)**. The optical densities were observed at 600 nm every 2 h. Growths were stopped at late-stationary phase, after 16 and 30 h, respectively for medium containing glucose or biphenyl.

KF707 W.T. and seven terminal oxidase mutant strains, namely: KFΔcox1-2 (Caa_3_ and Ccaa_3_ minus), KFΔcco1-2 (Cbb_3_1 and Cbb_3_2 minus), KFΔcox1-2/cco1-2 (Cbb_3_1, Cbb_3_2, Caa_3_, and Ccaa_3_ minus), KFΔCIO (CIO minus), KFΔcox1-2/CIO (Caa_3_, Ccaa_3_, and CIO minus), KFΔcco1 (Cbb_3_1 minus), and KFΔcco2 (Cbb_3_2 minus), were tested for their capacity to grow aerobically with glucose or biphenyl as sole carbon source. As shown in Figure 3, the KFΔcco1-2 and KFΔcox1-2/cco1-2 mutant strains were clearly impaired in their aerobic growth profiles and generation times (*g*) in glucose (*g* = 76 ± 1.5 and 88 ± 1.5 min, respectively; *p* ≤ 0.05) as compared to W.T. (*g* = 63 ± 0.8 min; *p* ≤ 0.05). Similarly, KFΔcco1-2 and KFΔcox1-2/cco1-2 mutant strains were shown to grow slowly in biphenyl (*g* = 140 ± 21 and 147 ± 26 min, respectively; *p* ≤ 0.05) as compared to W.T. (*g* = 97 ± 8.5 min; *p* ≤ 0.05). The results also indicated that the KFΔcox1-2 and KFΔCIO mutant growth curves were similar to KF707 W.T. growth curve regardless of the carbon source used for growth although a slight but significant increase of the generation time was seen in KFΔCIO cells grown in glucose and biphenyl (*g* = 75 ± 1.4 and 113 ± 1.5 min, respectively; *p* ≤ 0.05). The specific contribution of Cbb_3_1 and Cbb_3_2 to cell growth was also examined (Figure S1). The growth curves show that the lack of the Cbb_3_1 oxidase impaired the growth rate in both glucose (*g* = 85 ± 0.4 min; *p* ≤ 0.05) and biphenyl (*g* = 151±1.7 min; *p* ≤ 0.05) while the lack of Cbb_3_2 oxidase did not significantly affect the growth with both carbon sources (*g* = 75 ± 5 min and 110 ± 4.4, respectively). These results, taken together, indicate that the KF707 optimal growth rates in glucose or biphenyl depend on the simultaneous presence of both *cbb*_3_ type oxidases along with that of the CIO oxidase. This conclusion is also supported by evidence that despite numerous efforts, it was not possible to obtain a triple oxidase mutant lacking the CIO, Cbb_3_1, and Cbb_3_2 oxidases, so to suggest that this triple mutant was not viable under the tested aerobic growth conditions.

In addition to growth curves, KF707 W.T. and deletion mutant phenotypes were tested through the use of the NADI assay (Figure S2). This assay aims to visualize the cytochrome *c* oxidase respiration following the time-course oxidation of the artificial electron donor N,N,dimethyl-p-phenylenediamine (DMPD) which appears colorless in its reduced state or blue when it is oxidized to indophenol. With cells grown on glucose the assay was negative only in the case of KFΔcco1-2 and KFΔcox1-2/cco1-2 mutants, suggesting a negligible activity of *c(c)aa*_3_ type oxidases. On the contrary, in cells grown on biphenyl the assay was negative only in the case of KFΔcox1-2/cco1-2, suggesting a functional role of the *caa*_3_ type oxidase with biphenyl as the carbon source.

### Spectroscopic analysis and respiratory activities

Table 2 and Figure S3 summarize the heme-spectroscopic features of membranes isolated from cells grown in glucose as compared to those from cells grown in biphenyl and harvested at their stationary phase of growth. The spectroscopic evaluation of membranes from glucose grown cells showed that the α band attributable to *aa*_3_ type hemes is barely detectable at 600–605 nm. Conversely an evident peak at 600–605 nm which is attributable to *aa*_3_ type heme (Table 2) was seen in KF707 membranes from cells grown on biphenyl (Figure S3). This observation is therefore in line with the gene expression values reported in Figure 2B showing that the *cox1* promoter activity for the Caa_3_ oxidase is 4-times enhanced in KF707 biphenyl grown cells harvested in their stationary phase. The spectroscopic results of Table 2 also indicate that the protein subunits encoded by the gene clusters *cox123, coxMNOP, ccoN1O1Q1P1*, and *ccoN2O2Q2P2* (Table S2) all contain considerable amounts of *c*-type hemes as predicted by BLASTP search. Indeed, the amount of *c*-type heme as detected at 551–542 nm, decreased in KFΔcox1-2 and/or KFΔcco1-2 mutants and even more drastically in KFΔcox1-2/cco1-2 mutant cells (Figure S3).

**Table 2:** Heme amounts of *a*-, *b*-, and *c*-type in membranes isolated from KF707 W.T. and oxidase mutant cells grown with glucose and/or biphenyl as unique carbon source.

**Carbon source**	**Strain**	***c*-type heme e _551−540_ = 19.1 (nmoles/mg protein)**	***b*-type heme e _561−575_ = 22 (nmoles/mg protein)**	***a*-type heme e_603−630_ = 11.6 (nmoles/mg protein)**
GLUCOSE	KF707 W.T.	1.65 ± 0.20	0.77 ± 0.08	0.10 ± 0.05
	KFΔcox1-2	1.50 ± 0.20	0.80 ± 0.09	n.d.
	KFΔcco1-2	0.95 ± 0.15	0.62 ± 0.07	0.12 ± 0.05
	KFΔcox1-2/cco1-2	0.74 ± 0.10	0.64 ± 0.07	n.d.
	KFΔCIO	1.42 ± 0.15	0.84 ± 0.10	0.12 ± 0.05
	KFΔcox1-2/CIO	1.09 ± 0.12	0.61 ± 0.07	n.d.
BIPHENYL	KF707 W.T.	1.80 ± 0.20	0.87 ± 0.10	0.45 ± 0.03
	KFΔcox1-2	1.10 ± 0.09	0.82 ± 0.10	n.d.
	KFΔcco1-2	0.86 ± 0.07	0.59 ± 0.06	0.27 ± 0.02
	KFΔcox1-2/cco1-2	0.65 ± 0.08	0.32 ± 0.01	n.d.
	KFΔCIO	0.78 ± 0.09	0.50 ± 0.03	0.30 ± 0.02
	KFΔcox1-2/CIO	0.81 ± 0.07	0.55 ± 0.03	n.d.

*Symbols and abbreviations used: n.d., not-detectable by optical spectroscopy at room temperature. Values are the mean of at least two membrane preparations from different cell growth cultures for each strain. Mutant phenotype strains abbreviations as in Table 1. See Materials and Methods for experimental details*.

Oxidases of *aa*_3_ and *cbb*_3_ heme type are thought to function as cytochrome *c* oxidases whereas CIO was characterized as a quinol oxidase (Cramer and Knaff, 1990). In the past it was shown that both *cbb*_3_ and *aa*_3_ type oxidases in *R. sphaeroides* membranes were inhibited by cyanide (CN^−^) and azide (${\text{N}}_{3}^{-}$) anions (Daldal et al., 2001). However, while 50 μM CN^−^ fully inhibited both the *cbb*_3_ and *aa*_3_ type cytochrome *c* oxidase activities, 50 μM ${\text{N}}_{3}^{-}$ only inhibited the *cbb*_3_ dependent activities. Thus, in the presence of 50 μM cyanide or azide it was possible to determine the contribution of *aa*_3_ type and/or *cbb*_3_ type oxidases to the total respiratory activity catalyzed by cytochrome *c* oxidases (Daldal et al., 2001). Here, the same inhibitor concentration was used to estimate the activities of KF707 Cbb_3_ and C(c)aa_3_ oxidases in the presence or absence of cyanide and azide in membrane obtained from W.T. and mutant cells grown with glucose and biphenyl (Tables 3, 4).

**Table 3:** Respiratory activities in membranes from KF707 W.T. and oxidase mutant cells grown, until the stationary phase (OD_600 nm_) 0.7–0.9, in glucose (Gc) as sole carbon source.

**Electron donors**	**NADH**			**ASCORBATE**			
**Additions**	**/**	**${\text{N}}_{3}^{-}$**	**CN^−^**	**Cyt C**	**TMPD**	**TMPD/${\text{N}}_{3}^{-}$**	**TMPD/CN^−^**
**STRAINS**							
KF707 W.T.	165 ± 10	72 ± 3.3	29 ± 5.0	33 ± 1.0	157 ± 20	32 ± 3.0	7.0 ± 2.0
KFΔcox1-2	178 ± 20	38 ± 8.0	34 ± 10	59 ± 6.0	221 ± 14	21 ± 2.5	16 ± 1.0
KFΔcco1-2	175 ± 7.0	160 ± 2.0	125 ± 1.0	9 ± 0.5	38 ± 5.0	32 ± 3.3	8.0 ± 1.0
KFΔcox1-2/cco1-2	174 ± 15	172 ± 15	167 ± 15	1.0 ± 0.2	11 ± 2.0	11 ± 2.0	11 ± 2.0
KFΔCIO	145 ± 30	29 ± 5.0	1.0 ± 0.2	37 ± 3.5	208 ± 31	35 ± 3.5	10 ± 0.5
KFΔcox1-2/CIO	145 ± 5.0	25 ± 5.0	2.0 ± 1.0	45 ± 7.0	163 ± 12	10 ± 2.5	10 ± 2.5
KFΔcco1	196 ± 7.0	165 ± 2.0	110 ± 3.0	26 ± 2.5	124 ± 5.0	12 ± 3.0	3.0 ± 1.0
KFΔcco2	220 ± 10	78 ± 5.0	51 ± 5.0	33 ± 2.5	145 ± 5.0	37 ± 3.5	9.0 ± 2.0

*Symbols and abbreviations used: ${\text{N}}_{\text{3}}^{-}$, sodium azide (30 μM); CN^−^, potassium cyanide (50 μM); Cyt C, horse-heart cytochrome C (50 μM); TMPD, N,N,N′,N′-Tetramethyl-p-phenylene diamine (50 μM). Mutant phenotype strains abbreviations as in Table 1. Rates, expressed as nmoles of O_2_ consumed min^−1^ mg of proteins^−1^, are the mean of at least two/three membrane preparations from independent cell cultures*.

**Table 4:** Respiratory activities in membranes from KF707 W.T. and oxidase mutant cells grown, until the stationary phase (OD_600 nm_) 0.7–0.9, in biphenyl (Bp) as sole carbon source.

**Electron donors**	**NADH**			**ASCORBATE**			
**Additions**	**/**	**${\text{N}}_{3}^{-}$**	**CN^−^**	**CytC**	**TMPD**	**TMPD/${\text{N}}_{3}^{-}$**	**TMPD/CN^−^**
**STRAINS**							
KF707 W.T.	111 ± 2.5	64 ± 9.5	7.0 ± 0.3	37 ± 2.0	165 ± 13	88 ± 15	7.0 ± 2.0
KFΔcox1-2	38 ± 5.0	9.0 ± 3.3	3.0 ± 0.2	17 ± 3.0	75 ± 10	5.0 ± 2.0	3.0 ± 2.0
KFΔcco1-2	96 ± 9.3	80 ± 12	29 ± 0.4	18 ± 3.0	117 ± 12	108 ± 5.0	6.0 ± 1.7
KFΔcox1-2/cco1-2	36 ± 5.0	35 ± 5.0	35 ± 5.0	2.0 ± 1.0	6.0 ± 2.5	6.0 ± 2.5	6.0 ± 2.5
KFΔCIO	83 ± 4.0	60 ± 5.0	4.0 ± 2.0	37 ± 0.4	174 ± 1.0	110 ± 5.0	12 ± 1.0
KFΔcox1-2/CIO	104 ± 4.0	15 ± 2.5	3.5 ± 1.5	8.3 ± 3.5	67 ± 1.0	9.3 ± 2.0	8.3 ± 2.0
KFΔcco1	111 ± 8.0	97 ± 7.0	37 ± 2.0	20 ± 2.5	201 ± 18	150 ± 15	20 ± 7.0
KFΔcco2	95 ± 5.0	46 ± 5.0	11 ± 3.0	25 ± 3.0	200 ± 20	114 ± 13	13 ± 5.0

*Symbols and abbreviations used: ${\text{N}}_{\text{3}}^{-}$, sodium azide (30 μM); CN^−^, potassium cyanide (50 μM); CytC, horse heart cytochrome C (50 μM); TMPD, N,N,N′,N′-Tetramethyl-p-phenylene diamine (50 μM). Mutant phenotype strains abbreviations as in Table 1. Rates, expressed as nmoles of O_2_ consumed min^−1^ mg of protein^−1^, are the mean of at least two/three membrane preparations from independent cell cultures*.

Table 3 shows that with NADH as an electron donor, the total oxygen consumption by KF707 W.T. membranes from cells grown in glucose (hereafter named Gcm) was 66% inhibited by 50 μM azide (contribution of *cbb*_3_ oxidases) while only a further 17% was inhibited by 50 μM cyanide [contribution of C(c)aa_3_ oxidases]. The NADH oxidation when measured in membranes from biphenyl grown cells (hereafter named Bpm) (Table 4), was 42% inhibited by 50 μM azide while a further 52% of the total respiration was sensitive to 50 μM cyanide. Apparently, the % contribution to the NADH respiration of C(c)aa_3_ oxidases that are resistant to 50 μM azide but sensitive to 50 μM cyanide, was 3-fold higher in Bpm (52%) than in Gcm (17%). In line with this, the ascorbate/TMPD oxidase activity which indicates the overall oxygen consumption catalyzed by cytochrome *c* oxidases, was 80 and 47% repressed by azide in Gcm and Bpm, respectively, confirming the main role of *cbb*_3_ type oxidases in Gcm but not in Bpm. Results in W.T. membranes were confirmed by activities determined in membranes from oxidase mutant cells, with some additional finding, namely: the lack of both Cbb_3_1 and Cbb_3_2 oxidases (KFΔcco1-2 mutant) resulted into a strong activation of CIO dependent activity in Gcm (72% resistant to CN^−^). Further, the CIO activation resulting from the lack of *cbb*_3_ oxidases was less evident in Bpm (30% resistant to CN^−^) in which 83% of the NADH activity was insensitive to azide confirming the main functional role of *caa*_3_ type oxidase using biphenyl carbon source (see also Figure 2). Accordingly, in Bpm-KFΔcco1-2 the ascorbate/TMPD oxidation was only 8% inhibited by azide while 95% was sensitive to cyanide (see also Figure S2, NADI assay). These data, taken together, allowed us to determine in Gcm-KFΔcox1-2 and Bpm-KFΔcco1-2 the IC_50_ for cyanide of Cbb_3_ and C(c)aa_3_ oxidase activities which were in the order of 4.10^−7^ M CN^−^ and 5.10^−6^ M CN^−^, respectively (Materials and Methods and Figure S4).

As expected by the preceding results, both activation and functional role of CIO in supporting NADH dependent respiration in Gcm was also seen in the quadruple oxidase mutant KFΔcox1-2/cco1-2 whose respiratory activity was 100% insensitive to 50 μM cyanide; further, the latter oxidative activity was catalyzed by CIO with a rate similar to that performed by W.T. membranes in the absence of inhibitors. Bpm-KFΔcox1-2/cco1-2was therefore used to determine the IC_50_ for cyanide of CIO to be close to 1 mM CN^−^ (Figure S4).

To better understand the lack of which of the two Cbb_3_ oxidases determines an activation of the CIO oxidase activity, and the contribution of each Cbb_3_ oxidase to the total respiration, the oxygen consumptions in membranes from KFΔcco1 (Cbb_3_1 minus) and/or KFΔcco2 (Cbb_3_2 minus) mutant cells grown in glucose or biphenyl, were determined. Results in Table 3 (bottom lines) show that in glucose grown cells the CIO catalyses 56% of the total respiration of KFΔcco1, being 3–4 times higher than the corresponding activity measured in KFΔcco2 and W.T. membranes, with a parallel minor role (16%) of the Cbb_3_2 oxidase. Differently, in KFΔcco2 membranes the Cbb_3_1 oxidase catalyses 65% of the total respiration compensating the low CIO activity. Accordingly, the β-galactosidase assays performed in KFΔcco1CIOLac and KFΔcco2CIOLac cell extracts indicated that the expression of CIO in KFΔcco1 increases 4 times as compared to that of KFΔcco2 (Figure S5). Interestingly, results in Table 4 (bottom lines) show that in biphenyl grown cells the CIO catalyses 33% of the total CN^−^ resistant NADH respiration of KFΔcco1 with a parallel minor role of the Cbb_3_2 oxidase (13%, see also Figure S5) and a prevalent contribution of the Caa_3_ oxidase (54%) to respiration. Notably, in KFΔcco2 the CIO activity decreased to 13% of the total NADH oxidation with a parallel increase of the contribution to respiration of Cbb_3_1 (43%) and Caa_3_ (43%) oxidases.

One consideration arising from the results of Tables 3, 4 concerns the low values obtained from measurements of cytochrome oxidase activities using horse-heart cytochrome *c* (HHCyt *c*) as electron donor. This activity, in either Gcm or Bpm was ~5–6 times lower than that measured with TMPD as electron donor suggesting the low capacity of soluble HHCyt *c* to reduce KF707 respiratory oxidases which are featured by *c*-type hemes as catalytic subunits. This finding contrasts with the capacity of HHCyt *c* to replace the soluble mono-heme cyt *c*_2_ which is the physiological electron donor to either *aa*_3_ or *cbb*_3_ type oxidases in *R. sphaeroides* (Daldal et al., 2001). Conversely, our observation in KF707 is in line with an early report in which it was shown that HHCyt *c* was a poor substrate for the *cbb*_3_ type oxidase activity of *V. cholera*. In this latter species, the rate of oxygen reduction with HHCyt *c* was several fold lower than with the soluble di-heme cyt *c*_4_ which was identified as the physiological electron donor to the Cbb_3_ oxidase (Chang et al., 2010) (see Discussion).

## Discussion and conclusions

### The branched respiratory chain of *P. pseudoalcaligenes* KF707

#### Terminal cytochrome oxidases

In the past, the heme-copper oxygen (HCO) reductases were classified into three families: (1) type A or mitochondrial like oxidases of *aa*_3_ type; (2) type B or *ba*_3_ type oxidases; and (3) type C or *cbb*_3_ type oxidases which are only detected in bacteria (Sousa et al., 2012). Additionally, cyt *bd* type oxidases which are phylogenetically unrelated to HCO, represent a second major superfamily (Jünemann, 1997; Borisov et al., 2011) functioning as quinol oxidases.

Type A oxidases encoded by the *cox* gene cluster are present in a wide range of bacteria and they have a high proton-pumping activity (Brzezinski et al., 2004; Arai et al., 2014). In general, *aa*_3_ type oxidases show a low affinity for oxygen and usually play a dominant role under high-oxygen conditions in bacteria such as *R. sphaeroides* and *B. subtilis* (Gabel and Maier, 1993; Winstedt and von Wachenfeldt, 2000; Arai et al., 2008). If we extend these widely accepted biochemical concepts to *P. pseudoalcaligenes* KF707 redox chain the peculiarity of the membrane terminal oxidase content of this BCPs degrader becomes apparent. In fact, while the sequence analysis allowed classifying Cox in the type A subfamily, the presence in the predicted subunit II (CoxM) of an uncommon extra C-terminal domain carrying two *c*-type hemes binding consensus sequences, suggests that this protein is a *ccaa*_3_ type HCO. In agreement to this the C-terminal extension of subunit II (CoxB) from the facultative anaerobic proteobacterium *S. oneidensis* MR-1 was shown to bind two *c* type hemes (Le Laz et al., 2014). Similarly, this unusual feature was reported in *Desulfovibrio* spp. (Lobo et al., 2008) and some species of the genus *Psychromonas, Colwellia*, and *Methylosarcina* (Le Laz et al., 2014). In *Shewanella* MR-1, the Ccaa_3_ oxidase was expressed at a low level but only under O_2_-rich growth conditions in LB-medium (Le Laz et al., 2014) while in KF707 the Ccaa_3_ oxidase is always expressed at a low level regardless the cell growth phase (Figure 2).

As opposed to the CoxM subunit, the CoxB subunit II of *T. thermophilus, B.subtilis*, and *R. marinus* has an extra domain carrying only one *c*-type heme (Lauraeus et al., 1991; Mather et al., 1991). This is the case of *caa*_3_ type oxidase of KF707 which is highly expressed during the stationary phase in biphenyl (Figure 2). In KF707, the presence of *c*-type hemes in the oxidase catalytic subunits is not only predicted by the amino acid sequences (Supplementary Material—Alignment 1) but it was supported by spectroscopic analysis in which a decrease of the *c*-type heme content in membranes from KFΔcox1-2 mutant (Caa_3_/Ccaa_3_ minus) is seen (Figure S3; Table 2).

Oxidases of *cbb*_3_ type normally show a very high affinity for oxygen and low proton-translocation efficiency. In bacteria such as *P. denitrificans, R. sphaeroides*, and *R. capsulatus, cbb*_3_ oxidases are known to be induced under low oxygen conditions (Preisig et al., 1996a; Mouncey and Kaplan, 1998; Swem and Bauer, 2002). In *P. aeruginosa* PAO1 one of these *cbb*_3_ type oxidases, Cbb_3_1, is constitutively expressed and plays a primary role in aerobic growth irrespective of oxygen concentration; on the contrary, the expression of Cbb_3_2 varies under low oxygen conditions or at the stationary growth phase (Kawakami et al., 2010). The latter regulatory mechanism is also present in KF707 grown on glucose or biphenyl as clearly demonstrated by the constitutive expression of Cbb_3_1 while the Cbb_3_2 expression varies as a function of the carbon source and growth phase being repressed in the stationary phase of growth in biphenyl (Figure 2).

CIO oxidases are copper-free enzymes that are insensitive to millimolar concentration of cyanide and they function as quinol:oxygen oxidoreductases. Direct determination through the use of Q-electrodes of the Q-pool redox state in membranes from aerobically grown *R. capsulatus* endowed with Cbb_3_ and CIO oxidases, indicated that the quinol oxidase of CIO type starts being involved in respiration when the Q-pool reduction level reaches ~25% (Zannoni and Moore, 1990). If this observation is applied to analogous bacterial respiratory chains as those of *R. sphaeroides, P. aeruginosa* and KF707 it is apparent that CIO pathways operate as redox valves to prevent the Q-pool from exceeding the 25–50% oxidation-reduction level that is the optimum Q-pool redox state to warrant an efficient energy transduction by the respiratory chain (Klamt et al., 2008). This would explain why cytochrome Cbb_3_ oxidases are prone to sense environmental redox changes and this is why *cioABC* genes coding for CIO, are up-regulated by deletion of the constitutive Cbb_3_1 oxidase in *P. aeruginosa* (Comolli and Donohue, 2004). Interestingly, also in *P. pseudoalcaligenes* KF707 the CIO promoter is up-regulated by deletion of the Cbb_3_1 isoform (this work Figure S5) as also confirmed by respiratory activities measured in membranes from Cbb_3_1 minus cells (KFΔcco1 mutant) in which the CIO pathway catalyzes 60% of respiration as compared to only 17% of W.T. cells (Table 3). This result confirms that CIO has a minor role in KF707 respiration unless the Cbb_3_1 isoform is lacking.

### The cyt bc1 complex and soluble c-type cytochromes

As shown in the past (Di Tomaso et al., 2002), both NADH- and succinic-dehydrogenases channel their electrons into a quinol/cytochrome *c* oxido-reductase complex (also referred to as complex III or cyt *bc*_1_ complex) which contains *b*- and *c*-type heme subunits coded by 1,212 and 780 bp genes, respectively (BAU72674; BAU72675) while the ORF coding for the Rieske domain iron sulfur, [2Fe-S] reductase subunit of the *bc*_1_ complex, is coded by 591 bp gene (BAU72673). The latter genome annotation confirms early data indicating that the NADH-dependent respiration in KF707 is inhibited by the antibiotic antimycin A which is a specific inhibitor of the cyt *bc*_1_ complex at the heme *b*_H_-Qi interaction site level (Cramer and Knaff, 1990; Di Tomaso et al., 2002).

**Figure 4:**
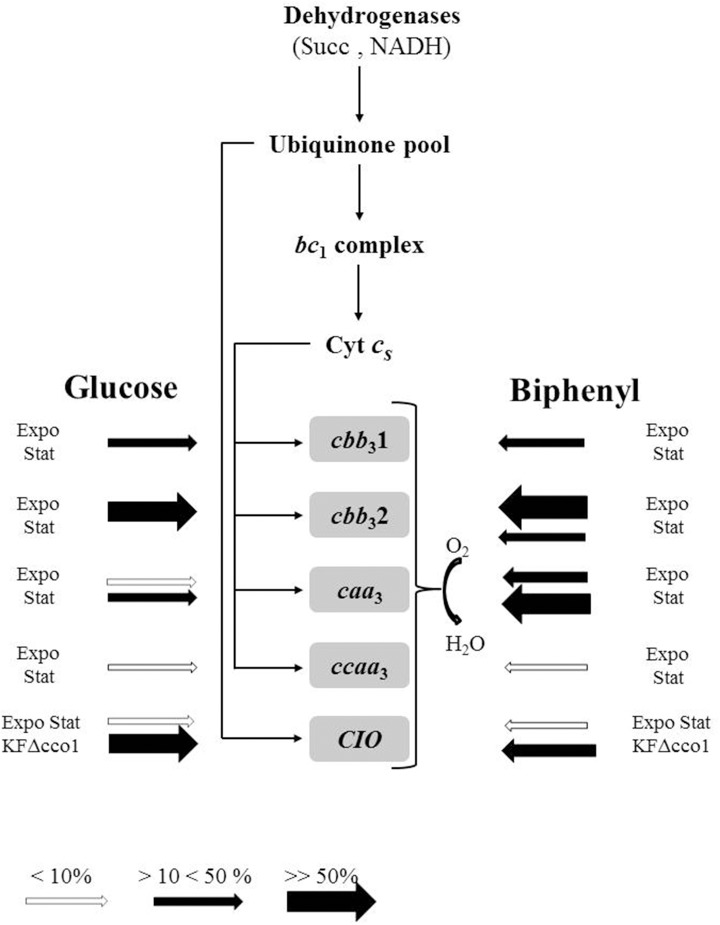
Block scheme illustrating the putative chain of KF707 based on spectroscopic, functional, and genetic analysis (this work and Di Tomaso et al., 2002). Symbols used: Succ, succinate dehydrogenase; NADH, NADH dehydrogenase; Cyt *Cs*, soluble cytochrome(s) *c*; *bc*_1_ complex, cytochrome *bc*_1_ complex III; CIO, cyanide insensitive oxidase (*bd* type) (encoded by *cioABC*); *caa*_3_, cytochrome *c* oxidase (encoded by *coxI-II-III* cluster); *ccaa*_3_, cytochrome *c* oxidase (encoded by *coxMNOP*); *cbb*_3_1, cytochrome *c* oxidase (encoded by *ccoN1O1Q1P1*); *cbb*_3_2, cytochrome *c* oxidase (encoded by *ccoN2O2Q2P2*); KFΔcco1, Cbb_3_1 minus mutant. The size and color of the arrows symbolize the % level of expression of terminal oxidases under the tested growth conditions, namely: cells grown in glucose or biphenyl and harvested during their exponential (Expo) or stationary (Stat) phase of growth. The actual expression values are those of Figure 2.

Analysis of KF707 annotated genome indicated the presence of genes encoding for soluble *c*-type cytochromes, namely: three genes identified as cyt *c*_4_ (BAU71765, BAU75530, and BAU75507) and two genes identified as cyt *c*_5_ (BAU77240 and BAU71764) (Supplementary Material—Table S3). Genes BAU71765 and BAU71764 coding for *c*_4_ and *c*_5_, respectively, are located in tandem on the same operon (not shown). In the working scheme of Figure 4 (see below) these two soluble *c*-type cytochromes, collectively named as Cyt *c*_s_, are supposed to function as electron donors to KF707 terminal oxidases. Because the aim of the present study was to analyse the role of the respiratory oxidases no further effort was made to understand the specific function of the soluble *c*-type hemes in KF707.

### Effect of the carbon source on the arrangement of KF707 respiratory chain

KF707 cells grown in LB-medium do not contain detectable amounts of *aa*_3_-type hemes regardless the phase of growth (Di Tomaso et al., 2002). Conversely we show here that spectroscopic significant amounts of *c(c)aa*_3_ type hemes are present in KF707 cells grown in minimal-salt media supplemented with biphenyl as sole carbon source (Table 2). Further, results of Figure 2 and Table 4 indicate that both *cox123* gene expression and catalytic activity (Caa_3_ oxidase) are greatly enhanced in biphenyl grown cells. This effect was paralleled by a drastic decrease (7-fold) in the expression of the Cbb_3_2 oxidase which was not compensated by a parallel increase of the CIO quinol oxidase activity while the Cbb_3_1 oxidase was constitutively expressed (Figure 2 and Table 4). These results are of particular interest because they outline a new bioenergetics scenario in which two respiratory oxidases of *P. pseudoalcaligenes* KF707 are modulated by biphenyl that is the metabolite which allows the co-metabolic degradation of PCBs by KF707. In this respect, while the response of the central carbon catabolism to environmental signals such as oxygen and/or nutritional compounds has been analyzed in some detail (Arras et al., 1998; Krooneman et al., 1998), the modulation of the oxidative ETC by the carbon source used for growth is far less documented (Dominguez-Cuevas et al., 2006; Arai, 2011; Arai et al., 2014). It is known that oxidases of *aa*_3_ type are affected by carbon starvation which was shown to induce a *cox* gene up-regulation in *P. aeruginosa*. This regulatory mechanism is linked to a cell response toward a more efficient energy-transducing *aa*_3_ oxidase under low nutrient conditions (Kawakami et al., 2010; Arai, 2011). As far as concern the role of *c(c)aa*_3_ type oxidases in KF707 grown in biphenyl, the growth results can be summarized as following (Figure 3B): (1) the growth rate of the *c(c)aa*_3_ double mutant (*g* = 94 ± 6.1 min) is similar to that of W.T. (*g* = 97 ± 8.5 min); (2) the lack of both Cbb_3_1 and Cbb_3_2 oxidases slows down KF707 cell growth (*g* = 140 ± 21 min); (3) the C(c)aa_3_/CIO minus phenotype is only slightly affected in its growth rate (*g* = 117 ± 1.5 min) as compared to W.T.; (4) the Cbb_3_1 minus phenotype is impaired in its growth rate (*g* = 151 ± 1.7 min) in spite of Caa_3_ over-expression; (5) growth of the quadruple cytochrome oxidase mutant Cbb_3_1-2/C(c)aa_3_, although impaired, is still supported by the oxidase activity of CIO which is up-regulated (*g* = 146 ± 16 min), and finally (6) all attempts to obtain a KF707 mutant carrying a Cbb_3_1-2/CIO minus phenotype were unsuccessful. Overall these data suggest that the C(c)aa_3_ oxidases of KF707 are unable to sustain aerobic growth when they are present as the only terminal oxidases as it was noticed in *Shewanella* MR-1 whose genome is predicted to encode for a terminal Cox oxidase annotated as Ccaa_3_ oxidase (Le Laz et al., 2014). In the past it was shown that a mutant of *P. aeruginosa* PAO1 that lacked four terminal oxidase gene clusters except for the *cox* genes (strain QXAa) was unable to grow aerobically in LB (Arai et al., 2014). More recently, a suppressor mutant of QXAa (QXAaS2) that grew aerobically using only the *cox* genes for *caa*_3_ (formerly reported as *aa*_3_) was described, in which a mutation in the two-component regulator RoxSR was necessary for the aerobic growth of PAO1 in LB (Osamura et al., 2017). Apparently, the expression and function of bacterial cyt *c* oxidases of *C(c)aa*_3_ type under variable growth conditions is far from being fully examined (Brzezinski et al., 2004; Arai, 2011; Osamura et al., 2017).

The scheme in Figure 4 represents the state of knowledge on the functional arrangement of the ETC in the PCBs-degrader *P. pseudoalcaligenes* KF707. A study is currently underway to understand the ETC's regulation mechanism as a function of the two carbon sources here used for cell growth, glucose, and biphenyl, along with the search for growth conditions under which the Ccaa_3_ oxidases of KF707 is significantly expressed.

## Author contributions

SF and FS: conceived, designed, and performed part of the experiments, assisted in design of the study and co-wrote the Manuscritpt. MC: assisted in design of the study and co-wrote the manuscript. FC and RT assisted in design of the study. DZ: conceived, directed, supervised, and co-wrote the Manuscript.

### Conflict of interest statement

The authors declare that the research was conducted in the absence of any commercial or financial relationships that could be construed as a potential conflict of interest.
