# Supplementary material for: Biphenyl Modulates the Expression and Function of Respiratory Oxidases in the Polychlorinated-Biphenyls Degrader Pseudomonas pseudoalcaligenes KF707
Source: Front Microbiol. 2017 Jun 30;8:1223. doi: 10.3389/fmicb.2017.01223 (PMC5492768; doi:10.3389/fmicb.2017.01223)
Supplement: Supplementary file 1 [file DataSheet1.PDF]

Supplementary Information of

Biphenyl modulates the expression and function of respiratory oxidases in the polychlorinated-biphenyls degrader *Pseudomonas pseudoalcaligenes* KF707

[1,*]Federica Sandri, [1,*]Stefano Fedi, [1]Martina Cappelletti, [2,3]Francesco Maria Calabrese, [4]Raymond J. Turner and [1,#]Davide Zannoni

[1]Department of Pharmacy and Biotechnology, University of Bologna, Bologna Italy

[2]Department of Biosciences, Biotechnology and Pharmacological Sciences, University of Bari "Aldo Moro," Bari, Italy

[3]Department of Biology, University of Bari "Aldo Moro", Bari, Italy

[4]Department of Biological Sciences, University of Calgary, Calgary,

Alberta, Canada, T2N1N4.

*These authors contributed equally to this work

[#] Corresponding author: Prof. Davide Zannoni, Via Irnerio 42, 40126 Bologna (Italy). Phone +390512091285 FAX +39051242576

email : davide.zannoni@unibo.it

Table S1: Primers for KF707 deletion and translational fusion mutants which were used in this study.

Target	Oligo nucleotide sequence (5'→ 3')	Enzyme site
**For cytochrome oxidases deletion mutants**		
**Δcox1**	UPFor ACGTGTAAGCTTCCTTCACAGTTATTCGGCGCA	HindIII
	UPRev GGAGTAAGTATGGAACAGTAGCGGCGGTGGATGATCATC	/
	DOWNFor ACTGTTCCATACTTACTCCTACCCAAGGCGGTGATCGAT	/
	DOWNRev ATTTCGAATTCTCTCGTCCAGCCGCGCTAAAG	EcoRI
**Δcox2**	UPFor CGGGCTGGATCCGCGAGACCC	BamHI
	UPRev ACATTGATCTTAATTGTACCTTCAGCCGGGAGCCGTCCG	/
	DOWNFor AGGTACAATTAAGATCAATGTCCTGCTTCCTCACCGGCTA	/
	DOWNRev ATCCCGAATTCTCACTCCCCTGCGCTACCAC	EcoRI
**Δcco1**	UPFor ATTAGTGGATCCAAGAGGACGGGGCGACGCAG	BamHI
	UPRev TAGGAGTCAGAATGGTTCAGTTAGGGGTTCCACGGTTAAT	/
	DOWNFor ACTGAACCATTCTGACTCCTAAAGTAACACCCCTGCCTGC	/
	DOWNRev GGGCTGAATTCGATGTAGAACTTGCGCTCGGG	EcoRI
**Δcco2**	UPFor AATCAGGATCCGACCCGAGGCTTGTCCGCTT	BamHI
	UPRev AGGAGTCAGTATGGAACAGTGTAGTTATAGGCGGTGCTGC	/
	DOWNFor ACTGTTCCATACTGACTCCTAAGAGGACGGGGCGACGCAG	/
	DOWNRev CTCCCGAATTCTAGGGGTTCCACGGTTAAT	EcoRI
**ΔCIO**	UPFor AATCAGGATCCGACTTACCTCAGCCCAAGG	BamHI
	UPRev TATCAGTCAGTATAGAACAGTATCTTCCTCGGTTACAGCG	/
	DOWNFor ACTGTTCTATACTGACTGATACCGGACACCACGCCCATGC	/
	DOWNRev CTCAAGAATTCCCAGGACTGCGGCGAGCC	EcoRI
**For lacZ traslational fusion mutants**		
**KFcox1Lac**	For TTTAAACTGCAGGTGGAGGCTGGCGACCTG	PstI
	Rev ATATCCAAGCTTAACAGACCACAGCAGCAG	HindIII
**KFcox2Lac**	For TTGTTAAGCTTGAGAGCCAGGATGATTGC	HindIII
	Rev TTATTGGATCCCCTTCGGCTTCGCCGC	BamHI
**KFcco1Lac**	For ATTACTAAGCTTAGCAGTCTGACTGATTGC	HindIII
	Rev ATTACGGATCCATTGCACCGCTTGCCACGG	BamHI
**KFcco2Lac**	For TTTCTGCAGCTTGTAGTTATAGGCGGTGTC	PstI
	Rev TTATTGGATCCCTGCTCGTGGGTGAAACCG	BamHI
**KFCIOLac**	For TAAAGGATCCTGCGGAACAGGGCATG	BamHI
	Rev ATACAAGCTTGTGGAAGGAGACCGTG	HindIII

Table S2: Description of KF707 cytochrome oxidases genes included in Fig. 1 as compared to PAO1 homologues. Names of the genes from which are encoded, lengths of the gene products in amino acids, and the Gene Bank accession numbers (*Pseudomonas pseudoalcaligenes* KF707 = NBRC 110670 – Nov. 2016), are reported. For each gene the Gene Bank accession number resulting as best hit from BLASTP against the *P. aeruginosa* PAO1 genome is reported with the related amino acids % of identity (aa % ID)

Oxidase gene cluster	Gene	Gene product (aa)	Gene Bank (accession no.)	aa % ID PAO1	Gene Bank (accession no.)
**Caa$_3$ (*cox1*)**	*coxI*	529	BAU71738	93%	AAG03496
	coxII	374	BAU71737	81%	AAG03495
	coxIII	295	BAU71740	85%	AAG03498
	*cox*11-*ctaG*	181	BAU71739	73%	AAG03497
	MFS	67	BAU71741	81%	AAG03499
	*surf*I	241	BAU71742	60%	AAG03500
	*cox*15-*ctaA*	361	BAU71744	77%	AAG03502
	*cox*10-*ctaB*	301	BAU71745	82%	AAG03503
	scoI	208	BAU71746	69%	AAG03504
**Ccaa$_3$ (*cox2*)**	*coxM*	481	BAU74428	26%	AAG03495
	coxN	588	BAU74429	42%	AAG03496
	coxO	229	BAU74430	29%	AAG03498
	coxP	227	BAU74431	37%	AAG03498
	coxX	105	BAU74432	/	/
**Cbb$_3$-1 Cbb$_3$-2 (*cco1-cco2*)**	*ccoN1*	480	BAU73555	90%	AAG04943
	ccoO1	203	BAU73554	94%	AAG04942
	ccoQ1	61	BAU73553	98%	ADN93060
	ccoP1	324	BAU73552	79%	AAG04941
	ccoN2	478	BAU73559	89%	AAG04946
	ccoO2	203	BAU73558	99%	AAG04945
	ccoQ2	55	BAU73557	70%	ADN93059
	ccoP2	311	BAU73556	72%	AAG04944
	ccoG	471	BAU73551	80%	AAG04940
	ccoH	165	BAU73550	57%	AAG04939
	ccoI	799	BAU73549	83%	AAG04938
	ccoS	73	BAU73548	74%	AAG04937
**CIO (*cio*)**	*cioA*	479	BAU72498	87%	AAG07317
	cioB	335	BAU72499	85%	AAG07316
	cioC	141	BAU72500	71%	AAG07315

Table S3. Genes predicted to encode cytochromes of c_4 and c_5 type in *P. pseudoalcaligenes* KF707 and comparison with the homologous genes found in *P. aeruginosa* PAO1 (Arai *et al.*, 2014).

Predicted gene product	KF707		PAO1		% ID[a]
	Accession no.	aa no.	Accession no.	aa no.	
cyt c_4	BAU71765	196	PA5490	201	85%
	BAU75530	218			34%
	BAU75507	218			34%
cyt c_5	BAU77240	140	PA5300	136	77%
	BAU71764	107	PA5491[b]	97	73%

[a]The % of amino acid identity as measured by pBLAST
[b]It was named as cyt c-552 by Matsuno and Yomoto (2015)

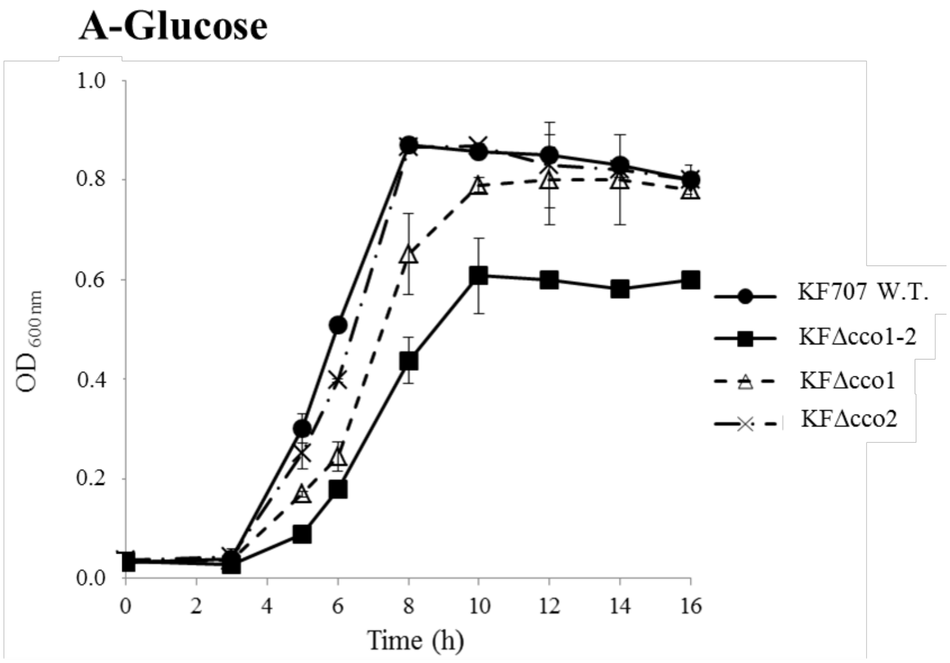

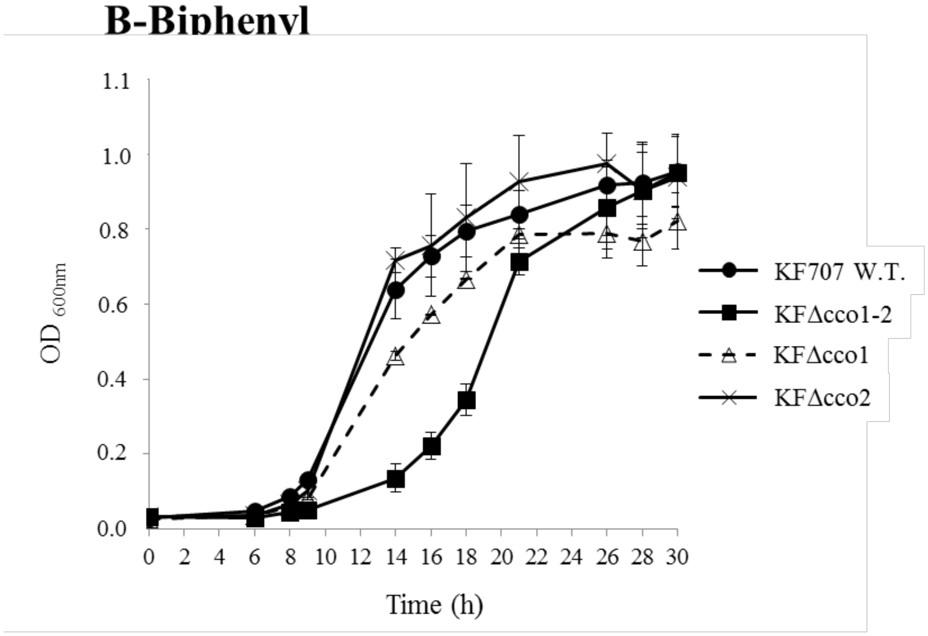

Fig S1 Growth curves of KF707 W.T. and deletion mutant strains (Table 1). Strains were grown in 50 ml of MSM medium in 250 ml flasks shaken at 130 r.p.m, with 6 mM of glucose (A) or biphenyl (B). The optical densities were observed at 600 nm every two hours. Growths were stopped at late-stationary phase, after 16 and 30 hours, respectively for medium containing glucose or biphenyl.

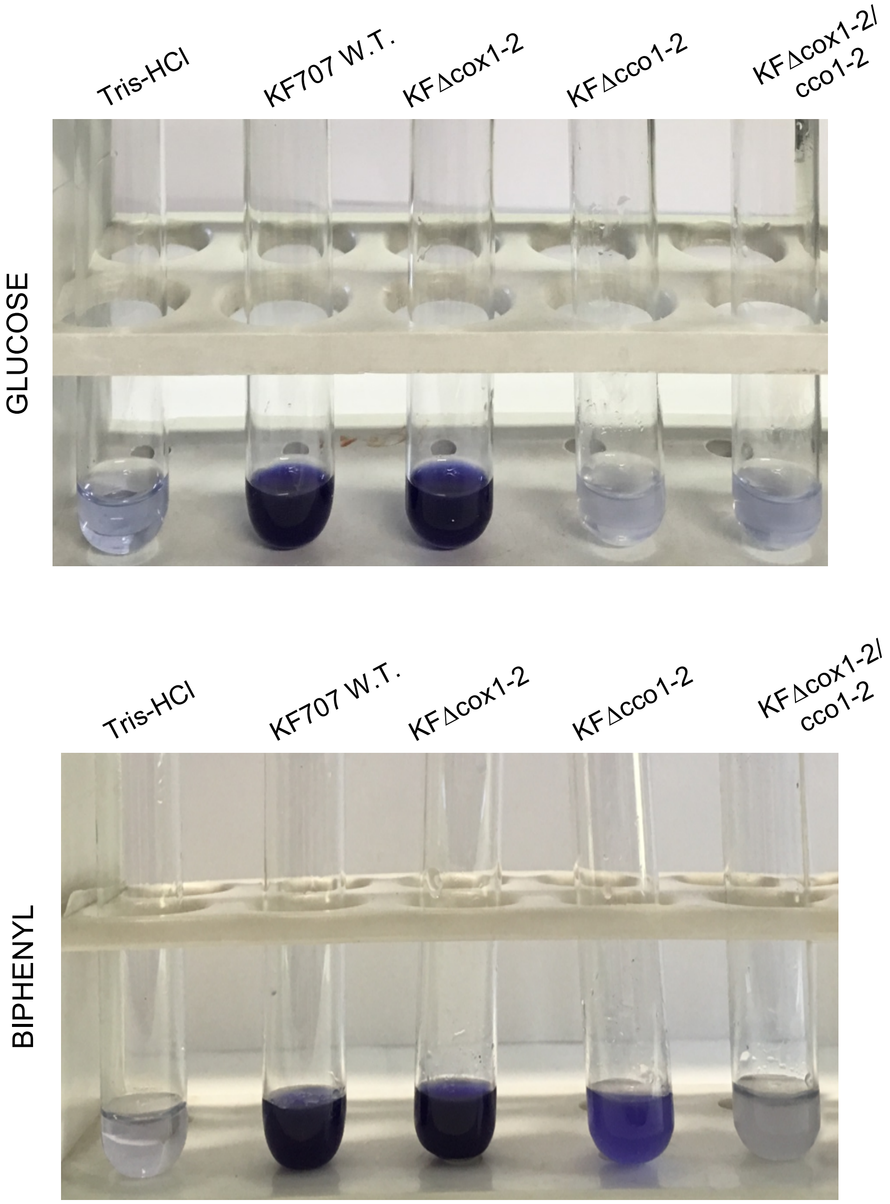

Fig. S2: Cytochrome *c* oxidase activity in KF707 wild type (W.T.) and deletion mutants strains (Table 1) as visualized in blue color by the NADI assay (Marrs and Gest, 1973). The assay was performed, after an overnight growth with glucose or biphenyl, with 1 mL of culture and 100 µl of a 1:1 mixture of 35 mM α-naphthol, in ethanol, and 30 mM *N,N*-dimetyl-*p*-phenylenediamine monohydrochloride (DMPD) in water.

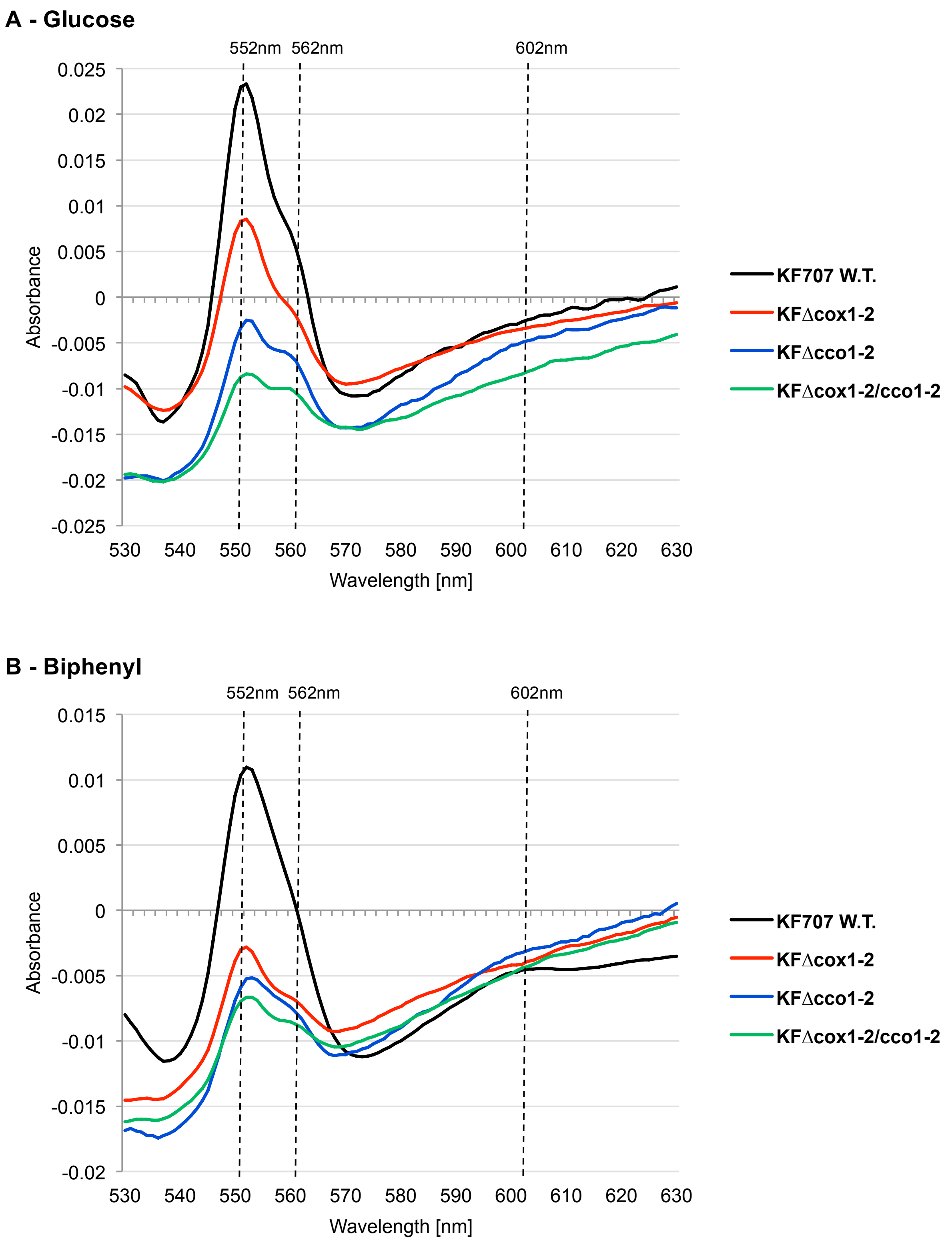

Fig. S3: Reduced-minus-oxidized difference spectra of membrane fragments from cells of KF707 W.T. and mutants grown with glucose and/or biphenyl as carbon source. Samples were oxidized with a few crystals of $K_4[Fe(CN)_6]$ and reduced with NADH (≅ 0,5 mM). Spectra were recorded from samples having the following membrane protein concentrations: Panel A - Glucose - KFΔcco1-2 and KFΔcox1-2/cco1-2, 11 mg ml^{-1}; W.T., 10.7 mg ml^{-1}; KFΔcox1-2, 11.9 mg ml^{-1}. Panel B - Biphenyl – KFΔcco1-2, 7 mg ml^{-1}; W.T., 8 mg ml^{-1}; KFΔcox1-2, 6 mg ml^{-1}; KFΔcox1-2/cco1-2, 7.2 m ml^{-1}.

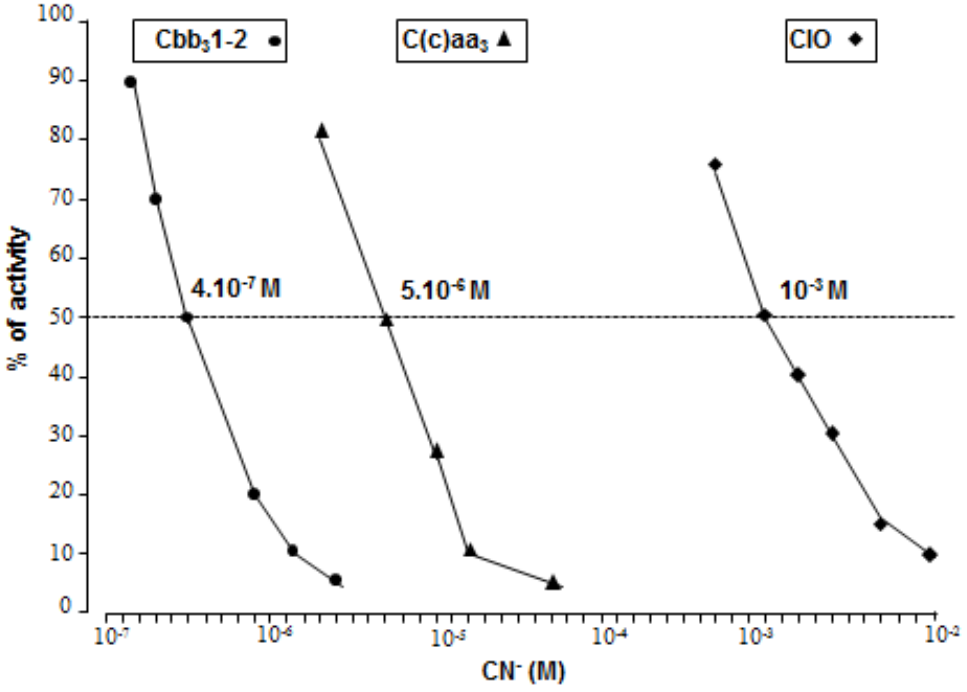

Fig. S4: Inhibition of the terminal oxidase activities in KF707 membranes. Cyt *c* oxidase catalyzed by Cbb_31-2 and $C(c)aa_3$ oxidases were determined as ascorbate/TMPD oxygen reduction in membranes of KFΔcox1-2 cells grown in glucose and KFΔcco1-2 cells grown in biphenyl, respectively. CIO oxidase was determined as NADH oxygen reduction in membranes of KFΔcco1-2/cox1-2 cells grown in glucose. The inhibition values are the mean of two independent membrane preparations. Continuous lines connecting the experimental points do not represent a mathematical function. The three values for CI_{50} CN^- are reported (see Materials and methods for details).

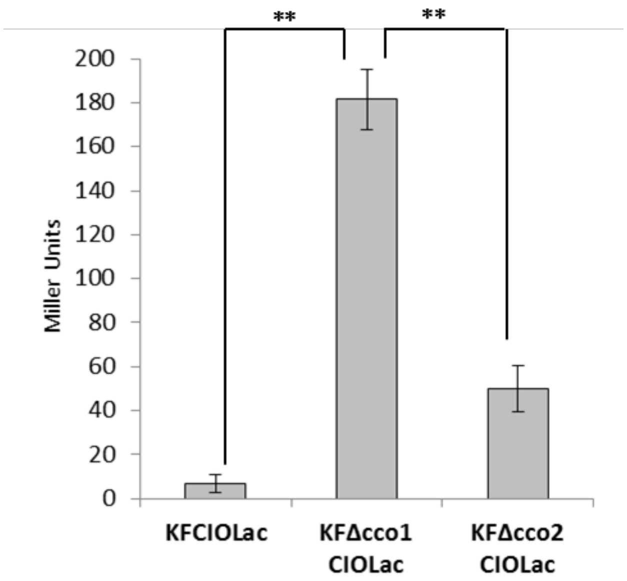

Fig. S5: β-Galactosidase activities measured in cell extracts derived from the KF707 translational fusion mutant strains (Table 1), grown aerobically in MSM medium with glucose as sole carbon source. The assays were performed six times and cells were harvested at their stationary growth phase (OD_{600nm} 0.7-0.9). Activities expressed in Miller Units. The results represent the mean based on at least six independent experiments. Error bars indicate standard deviations of the means. Asterisks indicate that mean values are significantly different by one-way ANOVA and verified by a two-sample T test within pairs of strains (** p<0.01).

Alignment 1: Alignment of Caa$_3$ oxidases from various bacterial species: KF707, *Pseudomonas pseudoalcaligenes* KF707, PAO1, *Pseudomonas aeruginosa* PAO1, THER, *Thermus thermophilus* and RHOD, *Rhodotermus marinus*. Residues that coordinate the *c*-type heme are indicated in black (C….CH…M).

```
KF707      -MRHPRVWMGLLLWS--VLSQAQAAWTVNMTPGATEVSRTVFDLHMTIFWICVVIGVIVF    57
PAO1       MLRHPRVWMGFLLLS--AISQANAAWTVNMAPGATEVSRSVFDLHMTIFWICVVIGVLVF    58
THER       ---MQRSFAALGLWGLSLAQEAHRVAITHP---GGSFNQEVAFLFPWVYFFSFLIFLVVA    54
RHOD       ------------MSGMILLQN--TAWLPEA---ASSIAPEVDSLFHFWTLVSAIIFIGVV    43
                       : .    .:   .    .     . ..   *  *.      .. :* : *

KF707      GAMFWSMII--HRRSTGQQPAHFHESTTVEILWTVVPFVILVLMAIPATKTLIDIYDTSE    115
PAO1       GAMFWSMIV--HRRSTGQQPAHFHESTTVEILWTVVPFVILVVMAVPATRTLIHIYDTSE    116
THER       GSLAYVTWKFRARPEDQEEPPQIHGNDRLEVVWTLIPLAIVFVLFGLTAKALIQVNRPIP    114
RHOD       GAMTFFVVRYRRRRRDE-VPEPVQEKKVVELAWIVVPTILVLIVFAWGFRVYIKMYTAPP    102
           *:: :        *      *  .: .  :*: * ::*  ::.::     :. *.:

KF707      SGLDVQITGYQWKWHYKYLGQDVEFFSNLATPSEQIHNKAPKDEHYLLEVDQPLVVPVGT    175
PAO1       PELDVQVTGYQWKWQYKYLGQDVEYFSNLATPQDQIHNRQAKDEHYLLEVDEPLVLPVGT    176
THER       GAMKVEVTGYQFWWDFHYPELGL------------------------RNSNELVLPAGV    149
RHOD       DAYEILVHGYQWYWEFEYPN-GV------------------------KTTNELHVPAGQ    136
              .: : ***: *.:.*   .:                       .  : * :*.*

KF707      KVRFLITAADVIHSWWVPALAVKKDAIPGFVNESWTRIEKPGLYRGQCTELCGKDHGFMP    235
PAO1       KVRFLITSSDVIHSWWVPAFAVKRDAIPGFVNEAWTKVDEPGIYRGQCAELCGKDHGFMP    236
THER       PVELEITSKDVIHSFWVPGLAGKRDAIPGQTTRISFEPKEPGLYYGFCAELCGASHARML    209
RHOD       PVKLRMTSADVIHSFYVPAFRVKQDVLPDRYSALWFEATKPGEYTVFCTEYCGTQHAGML    196
            *.: :*: *****::**.:  *:*.:*.  .     .  :** *   *:* ** .*. *

KF707      IVVEAKSQEDFAKWLAARKEETAKLKELTDKEWTLDELVARGDKVY-HTSCAACHQPEGQ    294
PAO1       IVVDVKPKAEFDQWLAKRKEEAAKVKELTSKEWTKEELVARGDKVY-HTICAACHQAEGQ    295
THER       FRVVVLPKEEFDRFVEAAKASPAPV------------ADERGQQVF-QQNCAACHGVARS    256
RHOD       AKVIVHPREEFEQWLESAGIPEDMP------------LAELGARLYREKACFSCHSIDGS    244
             *  .  : :* :::                       * ::: .  * :**     .

KF707      GMP----PMFPALKGSKIATGPKA--------------DHLNIVFHGKPGTSMAAFGKQL    336
PAO1       GMP----PMFPALKGSKIVTGPKE--------------HHLEVVFNGVPGTAMAAFGKQL    337
THER       MPPAVIGPE-LGLWGNRTSLGAGIVENTPENLK-AWIRDPA----GMKPGVKMPGFP-QL    309
RHOD       R---LVGPSFKGLYGSTRTFEDGTTAVADENYLRESILQPGAKIVQGYPNVMPASYA-SL    300
                  *   .* *.                        .         *..   .:  .*

KF707      SEVDIAAVITYERNAWGNNTGDMVTPKEVLALKQAESQ 374
PAO1       NEVDLAAVITYERNAWGNDDGDMVTPKDVVAYKQKQQ- 374
THER       SEEDLDALVRYLEGLKVEGFDFGALPKF---------- 337
RHOD       SEREVAALIEFIKQQQ---------------------- 316
          .* :: *:: : .
```

Alignment 2: Alignment of Ccaa$_3$ oxidases from various bacterial species: KF707, *Pseudomonas pseudoalcaligenes* KF707, MR-1, *Shewanella oneidensis* MR-1 and DES, *Desulfovibrio vulgaris*. Residues that coordinate the two c-type hemes are indicated in black (C—CH…M).

```
KF707      MAIAI-ILALILVASVLFHFLAPWHLTPPASNW-GSIDTTLLITLVITGVFFIAVVGFMV    58
MR-1       MKQWLYCLLVVLFAPPLAASDMRYNMTPGVTEISGKVYHLHMTILYICCAIGLVVFGVMI    60
DES        --------------------MYPQSLSPV-----QQVDLAFYVIFGVSAVMLLGITATML    35
                                    ::*       .:       : :  .: : : . *:

KF707      VAIIRFRHREGRRARYEPESRRLEWWLMVVTSLGIVGMLAPGLVVYSDFVRVPKDAYPLE    118
MR-1       YAMINHRKSKGAVASHFHESTKVEIAWTVIPFVILILMAIPATKTLIAMEDPSNADLTVK    120
DES        WFVWRYDHRRNPVATEIPGSVLAETAWTLIPTLIVMALFYYGWAGYKALRTVPADALEVG    95
             : .. : ..  *     *   *    ::  : :: :   .      :         :

KF707      VVAQQWQWAFRFPGQDGQLGRADVSWVDARNPLGLDPRDPHGQDDVLVRGNEVRLPLDRP    178
MR-1       VTGSQWKWHYSYFDQDIEFYSI-----LATPRPQIEGNEVKGEHYLLEVDKPLVLPVNRK    175
DES        VKARMWSWIFEYPNGKR--------------------------------SSVLYVPAGKP    123
           * .  *.* : : . .                                 .. : :* .:

KF707      VKVLLRSKDVLHNFYIPQIRGKMDMVPGMVSHFWFTPTLAGEYEILCAEFCGVGHFNMRG    238
MR-1       IRFLMTSEDVIHSWWVPDFAVKKDANPGFINEAWTRIDKPGIYRGQCAELCGKDHGFMPI    235
DES        VKLDMTSVDVIHSLYIPAFRIKMDTVPGMQTYAWFKTDGPGEFDILCAEYCGLKHANMLS    183
           ::. : * **:*. ::* :  * *  **: .  *      * :   *** **  *  *

KF707      KLQVEPAPAFEQWLATQPTFAQVLASAGAP------SQGGLIERGRQLADTHGCPACHSQ    292
MR-1       VVKALPEAEFEAWVKEQKQAADAAAQAAQAALSQNLSKEELMTQGEQV-YLGHCAACHQP    294
DES        VVKAVEPDEFRKWLESSE---------------------APGGKGRALLDAYGCISCHSL    222
            ::.     *. *:  .                         :*. :     * :**.

KF707      DGSQSLGPGWKDLYGREVQLAD----GSRLKADAAYLRESILDPRARLVQGYPPVMVPYT    348
MR-1       NGEGLKG-VFPHLKGSPIAMGPLGAH-----------------IEIVLNGKAGTAMQAFS    336
DES        DGSPGPGTTFKGLYGAERVVVLGDGSKRKVIVDEAYLRRALKDPNAELVEGFEPIMPSTE    282
           :*.   *  :  * *    :                        .  *       *

KF707      FS--QDELAALVAFIRSLSAVGQ-----------QE------------------------    371
MR-1       KQLTTQEIAAVITYERNAWGNNTGDAAQAKDVDAHKSGGTNSEPVATTQPPSTTDAPKAV    396
DES        GVVPEQDFEDMIAWF---------------------------------------------    297
                :::  ::::

KF707      -------------SGGAGNLVEQGEKLAQSLGCLACHSLDGSKGVGPSWKGLYGHPVTLA    418
MR-1       TEPIASVDPASLPTLTHEALMAEGEKVY-VTFCAACHQVTGA-GMPPAFPALAGSAIATG    454
DES        -------------MHGNGLTREEGRRLMEQEGCLSCHSTDGSVVAGPTFKNLWGSEVDVL    344
                                 :*.::     * :**.  *:    *::  * *  :

KF707      ---DGSQVEADAAYLRESVLAPAARLVQGYSPLMPAFT-PSDT-ELDALIAFIRSRADPD    473
MR-1       PSTN----HIDI------------VVQGKTGTAMQAFGKQLTPQQLAAVITYERNAWGNN    498
DES        VDGVPKRVKVDADYVRESIVAPQKKLSKGWDPLMPGYD-SFTPEQMEAMMDYMRSLSGTP    403
                   . *              :    .  * .:       :: *:: : *.  .

KF707      SDDQEQAP---------------       481
MR-1       TGDTVQPADIARHGK--------       513
DES        DKAPADASSPGLHGGTSPAGKTQ       426
                :
```
